# Exploiting synergistic effect of CO/NO gases for soft tissue transplantation using a hydrogel patch

**DOI:** 10.1038/s41467-023-37959-y

**Published:** 2023-04-27

**Authors:** Xiaoduo Tang, Jingyan Ren, Xin Wei, Tao Wang, Haiqiu Li, Yihan Sun, Yang Liu, Mingli Chi, Shoujun Zhu, Laijin Lu, Junhu Zhang, Bai Yang

**Affiliations:** 1 Joint Laboratory of Opto‐Functional Theranostics in Medicine and Chemistry, The First Hospital of Jilin University, Jilin University, Changchun, PR China; 2 Department of Hand and Podiatric Surgery, Orthopedics Center, The First Hospital of Jilin University, Jilin University, Changchun, PR China; 3 State Key Laboratory of Supramolecular Structure and Materials, College of Chemistry, Jilin University, Changchun, PR China

**Keywords:** Drug delivery, Biomedical materials, Biomedical engineering, Drug delivery

## Abstract

Autologous skin flap transplantation is a common method for repairing complex soft tissue defects caused by cancer, trauma, and congenital malformations. Limited blood supply range and post-transplantation ischemia-reperfusion injury can lead to distal necrosis of the flap and long-term functional loss, which severely restricts the decision-making regarding the optimal surgical plan. To address this issue, we develop a hydrogel patch that releases carbon monoxide and nitric oxide gases on demand, to afford a timely blood supply for skin flap transplantation during surgery. Using an ischemia-reperfusion dorsal skin flap model in rats, we show that the hydrogel patch maintains the immediate opening of blood flow channels in transplanted tissue and effective blood perfusion throughout the perioperative period, activating perfusion of the hemodynamic donor site. We demonstrate that the hydrogel patch promotes distal vascularization and long-term functional reconstruction of transplanted tissues by inhibiting inflammatory damage and accelerating blood vessel formation.

## Abstract (web summary)

Autologous skin flap transplantation is a common method to repair complex soft tissue defects. Here the authors develop a hydrogel patch that releases carbon monoxide and nitric oxide gases on demand, to afford a timely blood supply for skin flap transplantation during surgery, just improving the incidence of distal necrosis of the flap and reducing long-term functional loss.

## Introduction

Skin, muscle, fat, nerve, and other complex soft tissue defects could arise from tumor resection, trauma, and congenital malformations, thus leading to tissue atrophy and dysfunction. Autologous free flap transplantation is the primary method of reconstructive surgery used to repair such defects^1^, however, similar to other organ transplants, free flap transplants often suffer from common complications such as sufficient blood perfusion, tissue microcirculation disturbance, early ischemia-reperfusion injury (IRI), insufficient donor area, distal ischemic necrosis, etc^2–6^. These undesirable consequences result in an increased number of repeatedly surgeries for patients, coupled with extended hospital stay duration, and a substantial burden on family, social, health, and economic routine patient care.

The survival area of grafted skin flaps is directly determined by sufficient blood perfusion, patency of the main axial anastomosis artery, and microcirculation in the transplanted tissue during the perioperative period^7,8^. The skin arteries only have a limited blood supply range; thus, skin area beyond the working range of an artery in the grafted skin flaps will have insufficient blood supply. This ischemia is more severe when the adjacent perforating arteries are the choke connection^9–11^.

Hence, if the choke anastomoses between adjacent perforator arteries could be converted into true vascular anastomoses, the area of the donor graft would be greatly increased. Therefore, pre-intervention methods for flap transplantation are frequently conducted to reduce the resistance of the choke area, promote the transition from choke anastomosis to the true anastomosis, and eventually increase the blood perfusion and survival area of the flap. Current interventions include surgical delay, BOTOX injection, and physical expansion, among others. These strategies, however, fail to satisfy clinical needs of emergency surgery due to the long-term cost and complications caused by multiple operations^12–14^. Further, IRI during the flap transplantation process can trigger chronic inflammation, leading to non-functioning or early dysfunction of the graft^15,16^. Therefore, a clinically desired intervention method is to timely open choke vessels during emergency surgery, maintain sufficient blood perfusion of the transplanted flap for an extended period, and significantly reduce IRI during the transplantation.

Various gas molecules, such as NO, CO, and H_2_S, play multiple roles at physiological concentrations in the cardiovascular system^17,18^. NO is known to regulate vascular tone and blood pressure, as well as platelet aggregation and thrombosis^19^. Recent studies have suggested that NO might be involved in the pathogenesis of several cardiovascular diseases, such as atherosclerosis and hypertension^20^. CO is another ideal target molecule for approaches to prevent and reduce transplant-induced damage. CO can exhibit anti-inflammatory effects, relax blood vessels, control the proliferation of vascular smooth muscle cells and endothelial cells^21,22^. H_2_S is considered to activate the NO pathway by stimulating the enzyme nitric oxide synthase (NOS), leading to increase NO production^23^.

Increasing evidence indicates that gas molecules could exert a clear therapeutic effect in target organs through a variety of synergistic interactions. Nevertheless, gas molecules have adverse characteristics, such as dose dependence, short half-life, and pharmacokinetic cycle, which severely limits the effectiveness of target enrichment. Furthermore, the therapeutic dose safety window of these gas molecules is narrow, as low concentrations are ineffective, and high concentrations can cause apoptosis and cell cycle arrest^24^. Thus, controlling the synergistic effects of gas molecules and facilitating gradient release at regions of interest could have significant implications for flap transplant survival and long-term postoperative recovery of tissue and organ transplants. The dual gas system of CO and NO is essential for facilitating successful transplants. The release of CO in the early stage can synergia with NO, thereby avoiding the adverse effects of inhibited blood flow patency caused by a high concentration of NO^25^. Furthermore, low-dose, slow-release of NO locally and accurately maintains long-term blood supply patency, while avoiding short-term blood pressure variation. The vasodilator effect of CO is reported to be dependent on the presence of NO, and NO in turn enhances the cytoprotective effect of CO^26^.

**Fig. 1:**
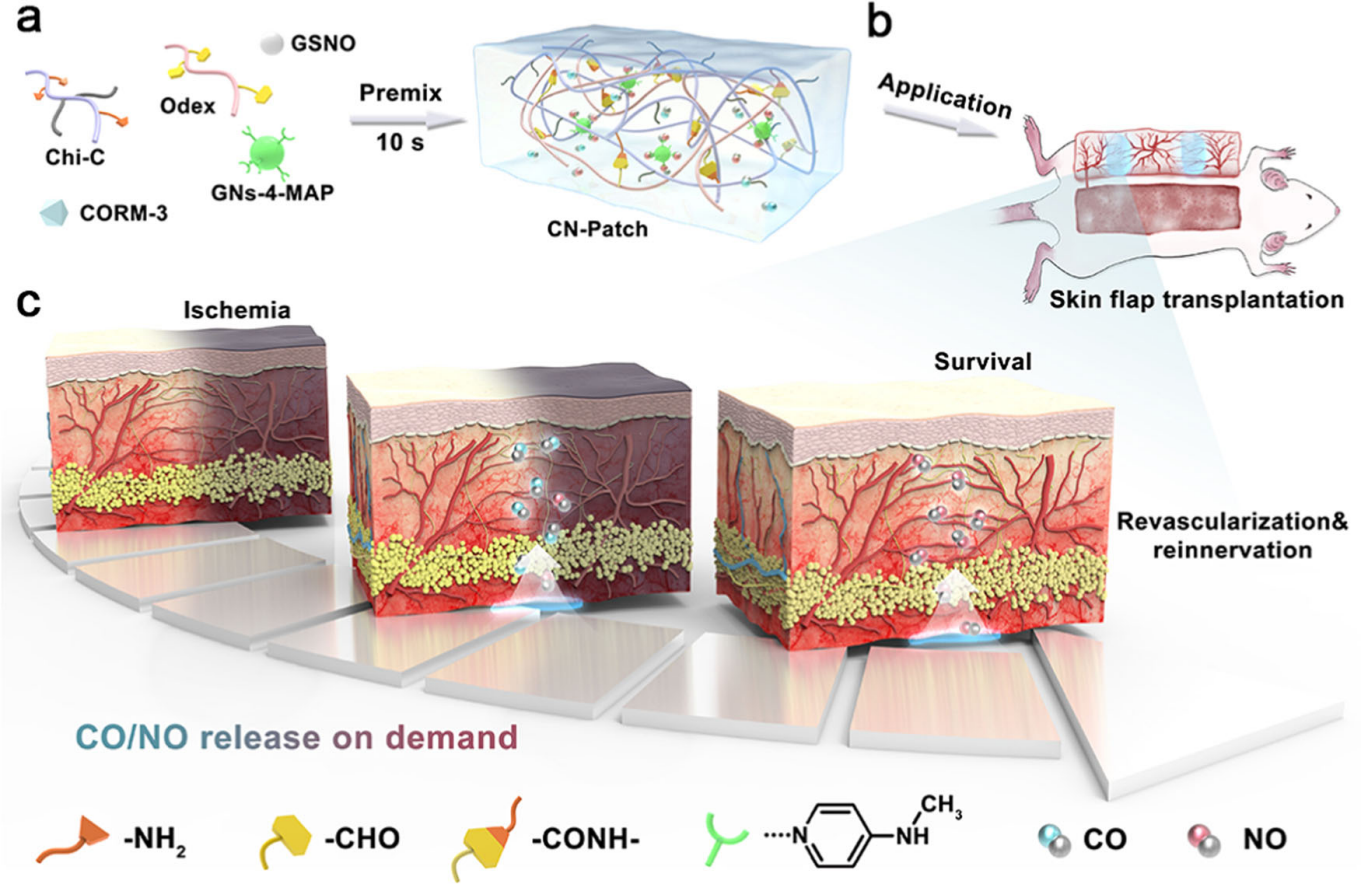
Schematic representation for the design strategy and application of CN-Patch hydrogel for revascularization of skin graft. **a** The schematic of the design strategy of the CN-Patch hydrogel. **b** The surgery flow chart of single pedicle ischemia flap model. **c** The ability of the CN-Patch to enable effective blood perfusion of transplanted tissue during early and long-term transplantation.

In this work, we develop a hydrogel patch for CO and NO gases release on demand (CN-Patch) (Fig. 1). The gelatin nanospheres (GNs) carrying 4-methoxy acetophenone (4-MAP, which can capture NO and extend the NO release period) (GNs-4-MAP) are next loaded into the gel. The classical CO/NO-releasing molecules Ru(CO)_3_Cl(glycinate) (CORM-3) and S-Nitrosoglutathione (GSNO) are selected as CO and NO donors, respectively. Using rat free flap transplantation and vascular anastomosis models, we verify the ability of the CN-Patch to enable effective blood perfusion of transplanted tissue during early and long-term transplantation.

## Results

### Preparation and characterization of the CN-Patch

**Fig. 2:**
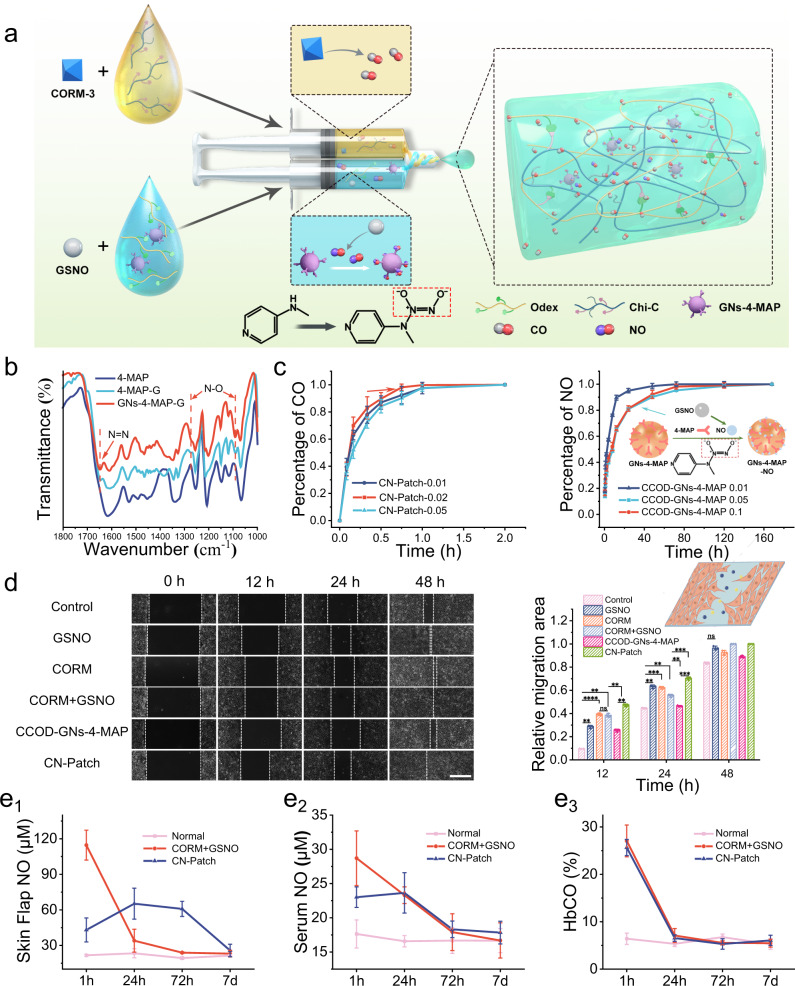
Characterization of the release effect of gas molecules in hydrogels. **a** The Schematic of the design strategy of the CN-Patch hydrogel. **b** The infrared spectra of the 4-MAP, 4-MAP-NO and GNs-4-MAP-NO, measured using a BLUCK spectrophotometer within 500–4000 cm^−1^. **c** CO and NO release study in vitro, measured using colorimetry and a NanoDrop Microvolume Spectrophotometer (ThermoFisher Scientific) (mean ± SEM, *n* = 6 samples per group). **d** The photo of (human umbilical vein endothelial cells) HUVECs cell migration. Scale bar: 500 μm. (Serum-free media were then utilized to halt proliferation for the evaluation of cell migration.) (mean ± SEM, *n* = 3 independent samples). **e** In vivo release of sustained release system in rats. e_1_, Determination of the skin flap NO concentration by colorimetry. e_2_, Serum NO levels. e_3_, Arterial blood carboxyhemoglobin saturation under different treatments. Error bars indicate Standard Error. The horizontal line indicates the Median Value. (mean ± SEM, *n* = 6 animals per group). Source data and exact *p* values are provided in the Source data file.

We manufactured a hydrogel patch, CN-Patch, for the controlled release of CO and NO gases (Fig. 2a). Inspired by previous investigations, our patch is composed of polysaccharides, which have been investigated for their suitability as a moisture barrier and for controlled release of therapeutic agents^27,28^. We first synthesized gelatin nanoparticles using a typical two-step desolvation method^29^. The 4-MAP was loaded into the GNs by utilizing the hydrogen bond interaction between 4-MAP and gelatin nanoparticles through a solution saturation lyophilization method^30,31^. Catechol derivatives modified chitosan (Chi-C) and aldehyde dextran (Odex) were used to synthesize a hydrogel patch (CCOD) with suitable tissue viscosity and hemostatic effect^30^. The physical-chemical characterization and biological safety evaluation of the CN-Patch are shown in Supplementary Figs. 1–8.

Further characterization of CN-Patch was necessary to determine its sustained gas release ability in vitro. The infrared spectra in Fig. 2b showed that, compared with the 4-MAP, 4-MAP doping with GSNO (4-MAP-G) and GNs-4-MAP doping with GSNO (GNs-4-MAP-G) had several new peaks at 1110 cm^−1^, 1272 cm^−1^, and 1650 cm^−1^ (stretching vibration of the N–O and N = N bonds), indicating successful loading of NO in the GNs-4-MAP^32^. To verify the feasibility of the CN-Patch delivery scheme, we further tested the release rate of CO and NO gas molecules. Results indicated that CORM-3 was rapidly hydrolyzed to release CO in the early stage (Fig. 2c). The release cycle of CO did not significantly change with the increase of the crosslinking density of the polymer due to its strong permeability to gas molecules. With the increase of GNs-4-MAP concentration, the sustained release period of NO was prolonged due to the increased number of NO capture sites. We thus selected a hydrogel monomer concentration of 2% and a GNs-4-MAP doping amount of 5% for forming the CN-Patch. Under these conditions, CO could be released in 0.5 h, and NO could be maintained for a sustained release period of 7 d, which met the therapeutic period of flap transplantation.

Due to limitations in accurately measuring the gas sustained release period, we further evaluated it using cell migration experiments (Fig. 2d). Due to the rapid hydrolysis, NO concentration was excessive in the GSNO group, which had an inhibitory effect on cells, while in the CORM, CORM + GSNO, and CN-Patch groups, rapid release of CO in the early stage significantly accelerated cell migration. Further, the cell migration rate of the CN-Patch group remained relatively fast at 24 and 48 h, consistent with the expectation that CN-Patch could release NO for extended periods.

To evaluate the concentration of gas molecules in vivo, we ground the skin flap of the normal, CORM + GSNO, and CN-Patch groups, and took the supernatant for measurement. The results in Fig. 2e_1_ showed that after local injection of CORM + GSNO solution for 1 h, the content of NO in skin flap tissues reached 5-fold higher than that of other normal tissues, and decreased rapidly to the normal level after 72 h. Conversely, in the CN-Patch group, the content of NO in skin flap tissues increased to twice than that of normal skin flap tissues within 1 hour, thrice after 24 h and remained for 72 h, and decreased to the normal level after 168 h. Collectively, these results demonstrated that CN-Patch had the effect to prolong the release time of NO.

We further collected rat femoral artery blood at different time points to detect the concentration of NO in serum (Fig. 2e_2_). The NO level in the serum of CORM + GSNO group was higher than that of CN-Patch group within 1 h, and became equivalent between these two groups after 24 h. This indicated that compared to the local injection of GSNO, the CN-Patch group had less disturbance to the NO cycle in vivo, which was more conducive to the precise release of the drug and the reduction of drug side effects.

The detection of carboxyhemoglobin saturation in serum in Fig. 2e_3_ revealed no significant difference between the CN-Patch group and the local injection of CORM + GSNO solution, which was consistent with the in vitro detection results. This was in accordance with our design goal of early release of CO against ischemia-reperfusion injury in transplanted tissues. Taken together, these results were in line with our design expectations.

### Multimodal flap blood supply monitoring to verify the effectiveness of CN-Patch

**Fig. 3:**
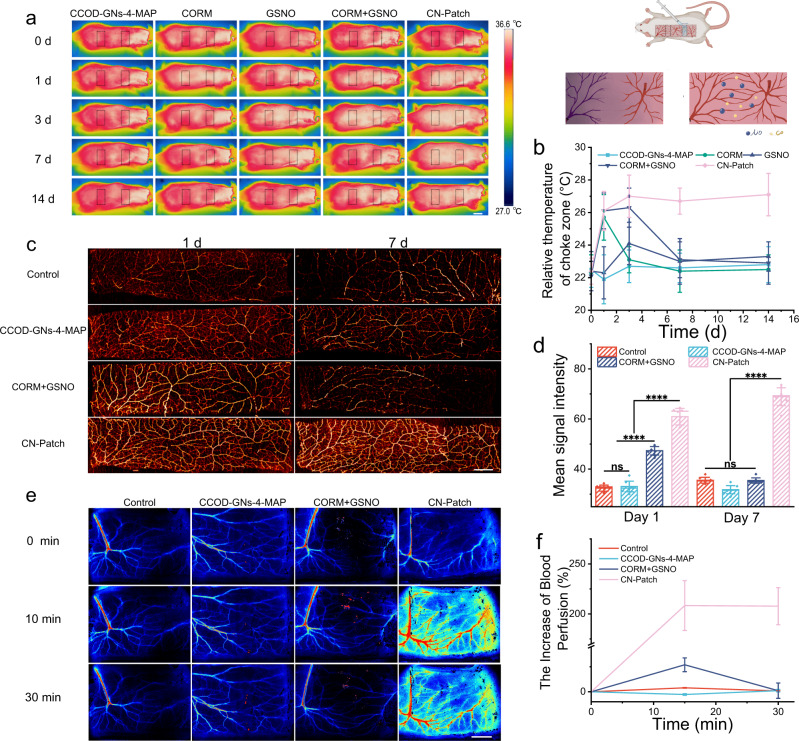
The vasodilating effect of CN-Patch. **a**, **b** The black rectangles indicated chokezones. The thoracic dorsal artery-posterior intercostal artery choke vascular area and the posterior intercostal artery-deep iliac circumflex artery choke vascular area were the key monitoring areas, and an infrared thermal imaging camera was used to track the local temperature after the intervention (days 0–14) (mean ± SEM, *n* = 3 independent animals per group). Scale bar: 1 cm. The top right illustration was designed using BioRender graphic tool (BioRender.com). **c** The postmortem arteriography of flaps on day 1 and day 7 after administration. Scale bar: 1 cm. **d** Mean signal intensity of arteriography images (mean ± SEM, *n* = 6 animals per group). **e** The laser speckle images of flap choke zones before and after administration. Scale bar: 1 cm. **f** The line graph of the increased blood perfusion ratio after the application of CN-Patch based on the laser speckle images (mean ± SEM, *n* = 3 independent animals per group). Error bars indicate standard error. The horizontal line indicates the Median Value. Source data and exact *p* values are provided in the Source data file.

A Standard 11 × 3 cm^2^ perforator flap, with three vascular pedicles, was generated on the backs of rats (Supplementary Fig. 9a). The infrared thermal imaging on the 14th day in Fig. 3a, b showed sufficiently sustained blood supply patency in the choke area of the skin flap compared to other groups, demonstrating that the CN-Patch could significantly prolong drug activity, inducing continuous expansion of blood vessels, and creating better conditions for the blood supply to the transplant after surgery. The CN-Patch could also potentially promote continuable vascular patency of the yoke vessels, thus permanently supplying blood to the graft tissue.

To further study the morphology of the blood vessels post-transplant, we performed perfusion contrast X-ray imaging of blood vessels in the flap area on the 1st and 7th day using Gelatin-PbO (an X-ray contrast agent). The density of blood vessels in the back flap areas of the CN-Patch was higher than the other three groups on the 1st and 7th day (Fig. 3c, d). This result revealed that the CN-Patch could induce the reestablishment of microvascular networks in the choke area and realize true anastomosis over a long-term period.

Based on the need for immediate intraoperative applications in clinical practice, a laser speckle was applied for real-time monitoring the blood supply in ischemia and reperfusion flaps of each group after 30 min (Fig. 3e, f; Supplementary Fig. 10; Supplementary Movie 1). Groups of CN-Patch and CORM + GSNO showed significant vasodilator effect at 15 and 30 min compared to other groups. The relative perfusion increment in the CN-Patch group was 7.4-fold higher than that of the CORM + GSNO group, and could be maintained for an extended period.

### Assessment of new blood vessel and nerve function

**Fig. 4:**
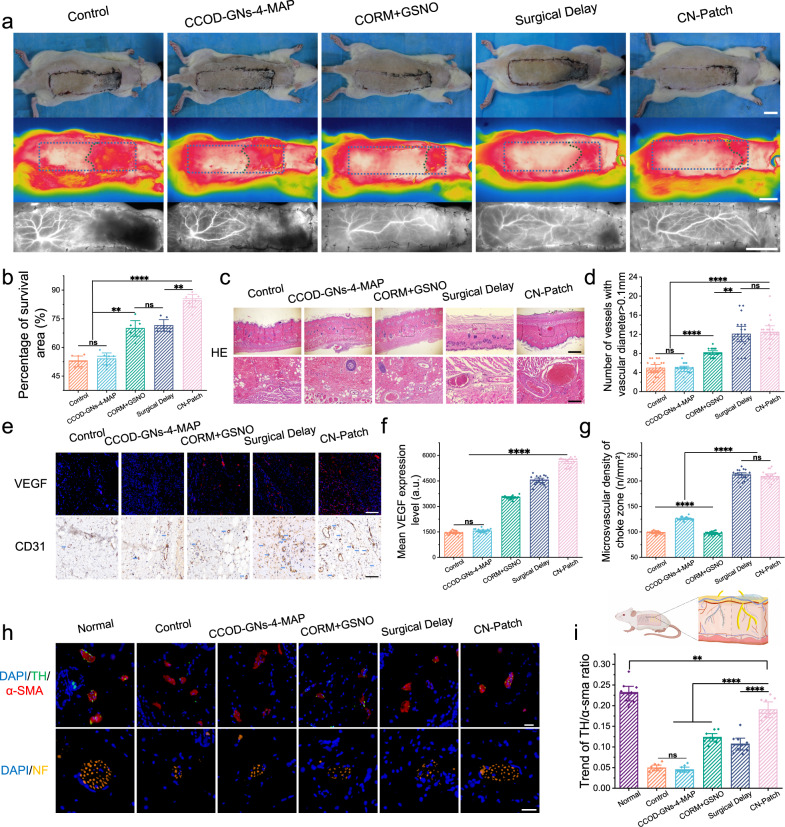
Survival status of flaps after treatment. **a** The first row of general observation, the second row of digital infrared thermal images, and the third row of NIR-II angiography show the survival rates of skin flaps on the seventh day after surgery. Scale bar: 2 cm. **b** The percentage of flap survival areas in each group on the postoperative seventh day (mean ± SEM, *n* = 6 animals per group). **c** First row, H&E staining of flap choke zones, black rectangles indicate dilated vessel. Scale bar: 200 µm. Second row, higher magnification of the images above. Scale bar: 50 µm. **d** The average number of vessels with a diameter greater than 0.1 mm in each group (mean ± SEM, *n* = 6 animals per group). **e** Distribution of VEGF (upper row, red) and CD31 (lower row) expression of choke zones on 7 d postoperative based on immunofluorescence and immunohistochemistry staining results. The blue arrows point to microvessels. Scale bar: 400 µm. **f** Mean VEGF fluorescence intensity (mean ± SEM, *n* = 6 animals per group). **g** Microvascular density of choke zone (mean ± SEM, *n* = 6 animals per group). **h**, **i** The immunofluorescent staining of tyrosine hydroxylase (green), α-SMA (red) label sympathetic neurons, and vascular smooth muscles of flap choke zones three months after surgery; immunofluorescence staining for neurofilament (NF, orange) expression in the flap nerve; nuclei were stained with DAPI (blue). Scale bar: 20 µm (mean ± SEM, *n* = 6 independent animals). Error bars indicate Standard Error. The horizontal line indicates the Median Value. Source data and exact *p* values are provided in the Source data file. This picture was designed using BioRender graphic tool (BioRender.com).

We next investigated flap necrosis rate and long-term repair effects. The survival areas of the flaps in the CORM + GSNO and CN-Patch groups were respectively 16.9% and 33.5% higher than that in the control group (Fig. 4a, b), indicating that the CN-Patch can effectively increase the flap survival area and open blood vessels in the choke area of the transplanted flap for an extended period, thereby reducing microcirculation disorders. To further explore the mechanism of vascular dilatation, we characterized the choke areas in the skin flaps of each group on the 7th day after surgery. We found that the density of neovascularization in the choke areas of flaps in the CN-Patch and CORM + GSNO groups was significantly increased relative to the control group (Fig. 4c, d). VEGF and CD31 expression levels were significantly increased in the CN-Patch and CORM + GSNO groups, and this was more obvious in the CN-Patch group (Fig. 4e–g). These data demonstrated that the CN-Patch can effectively induce endothelial cell proliferation and microvessel system remodeling, as well as significantly promote the revascularization of postoperative skin flaps^33,34^.

On the 90th postoperative day, we dissected the choke areas of flaps and stained them for neurofilament-200 (NF-200), α-smooth muscle actin (α-SMA), and tyrosine hydroxylase (TH) (Fig. 4h, i). The intercostal nerve and the dorsal thoracic nerve of the brachial plexus in the CN-Patch extended to the skin on the back, and the skin and blood vessels in the flaps of the CN-Patch group re-neuralized spontaneously. Immunohistochemical staining of the choke areas of flaps indicated that expression levels of IL-6 and TNF-α were both decreased in the CN-Patch group (Supplementary Fig. 11). The cutaneous trunci muscle reflex (CTMr) test showed that approximately 50% of sensory and motor activities were restored in skin flaps in the CN-Patch group (Supplementary Fig. 12)^35^.

### Assessment of blood supply in the vascular anastomosis area

**Fig. 5:**
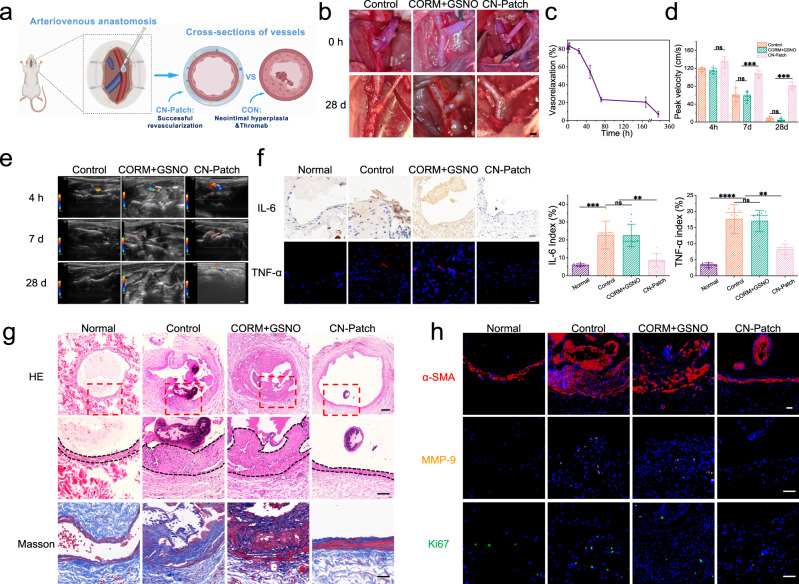
Application of the CN-Patch for improving vascular patency of the main blood vessel after arteriovenous anastomosis. **a**, **b** Establishment of the model of carotid artery to external jugular vein fistula in rats. All rats were then randomly divided into three groups, the CN-Patch was applied locally in the treatment group, the PBS group was deemed the control group, and CORM + GSNO was a drug solution group with PBS as the solvent. Scale bar: 1 mm. This picture was designed using BioRender graphic tool (BioRender.com). **c** The relaxation capacity of the right external jugular veins under leach liquors of the CN-Patch soaked in PBS was assessed using a DMT wire myograph system (DMT 620) for different periods (mean ± SEM, *n* =  3 independent samples). **d**, **e** The vascular patency was evaluated by visual observation (0 h, 28 d) and the ultrasound doppler (4 h,7 d, and 28 d), and the average peak flow velocity of the outflow vein was recorded (mean ± SEM, *n* = 6 independent samples). Scale bar: 2 mm. **f** Representative immunohistochemical staining of IL-6 and TNF-α of the venous outflow tracts 28 d post-surgery. Scale bar: 20 µm (mean ± SEM, *n* = 6 independent samples). **g** The representative images of HE and Masson staining of the venous outflow tracts 28 d post-surgery. Scale bar: 200 µm, 100 µm, and 100 µm from top to bottom. The neointimal index and the degree of fibrosis were calculated and analyzed (*n* = 6 independent samples per group). **h** Immunofluorescence staining of α-SMA (contractile phenotype of smooth muscle cells, red), MMP-9 (vasodilation phenotype of smooth muscle cells, yellow), and Ki67 (cell proliferation, green) of the venous outflow tracts were performed 28 d after the administration to further evaluate the degree of intimal hyperplasia, the percentage of the positive area of immunofluorescent staining were measured and calculated. Scale bar: 50 µm. (*n* = 6 independent samples per group). Error bars indicate Standard Error. The horizontal line indicates the Median Value. Source data and exact *p* values are provided in the Source data file.

The degree of blood flow patency of the main channel directly affects the outcome of organ transplantation; however, intimal damage, inflammation, hypoxia, and hemodynamic changes can inevitably lead to anastomotic restenosis, resulting in poor blood flow. The pedicle diameter of the deep axial iliac artery perforator flap in rats is only 0.2–0.3 mm, and the microanastomosis operation is random, leading to significant individual differences. Therefore, we selected the classic rat jugular arteriovenous fistula model to verify the function of the CN-Patch in maintaining main blood vessel patency (Fig. 5a, b). To explore whether adequate blood flow perfusion could likely be attributed to the direct vasodilation function of the CN-Patch, the relaxation capacity of external jugular veins was assessed in vitro. It was found that a single-dose of the CN-Patch was sufficient to maintain the vasodilation effect for more than 7 d (Fig. 5c). Ultrasound Doppler in the operation area after jugular arteriovenous anastomosis showed that the average peak flow velocity and patency of outflow veins in the CN-Patch group were significantly increased compared with those in the control group on the 4 h, 7th, and 28th days (Fig. 5d, e).

Pathological intimal hyperplasia and adverse vascular remodeling are thought to cause functional inactivation of anastomosis, and to assess the remodeling of the outflow vein^36^. Immunohistochemical staining of the venous outflow tracts 28 d post-surgery indicated that expression levels of IL-6 and TNF-α were both decreased in the CN-Patch group (Fig. 5f). H&E and Masson staining of the venous outflow tracts 28 d post-surgery revealed a significant decrease in the extent of intimal hyperplasia and fibrosis in the CN-Patch group compared to the control group (Fig. 5g). Semi-quantitative analysis showed that the average neointima/lumen area ratio in the CN-Patch group (0.132 ± 0.024) was significantly lower than that of the control group (0.224 ± 0.035). Simultaneously, the average neointimal area of blood vessels in the CN-Patch group was significantly reduced. Immunofluorescence staining of α-SMA, MMP-9, and Ki67 in the venous outflow tracts displayed synchronous decreases in the contractile and vasodilation phenotypes of smooth muscle cells after the CN-Patch administration (Fig. 5h). There was no significant difference between the CORM + GSNO group and the control group, which was due to the vascular anastomosis model being located in the neck with a larger cavity, making it difficult to achieve local enrichment and long-term effects of the drug after a single solution administration. These results suggested that adventitial application of the CN-Patch could reduce the formation of postoperative venous stenosis by inhibiting smooth muscle cell proliferation with anti-thrombotic and anti-inflammatory effects, further confirming the ability of the CN-Patch to maintain main channel patency following graft vessel anastomosis (Supplementary Fig. 13).

### The CN-Patch improves transplant survival by promoting angiogenesis and reducing inflammatory responses

**Fig. 6:**
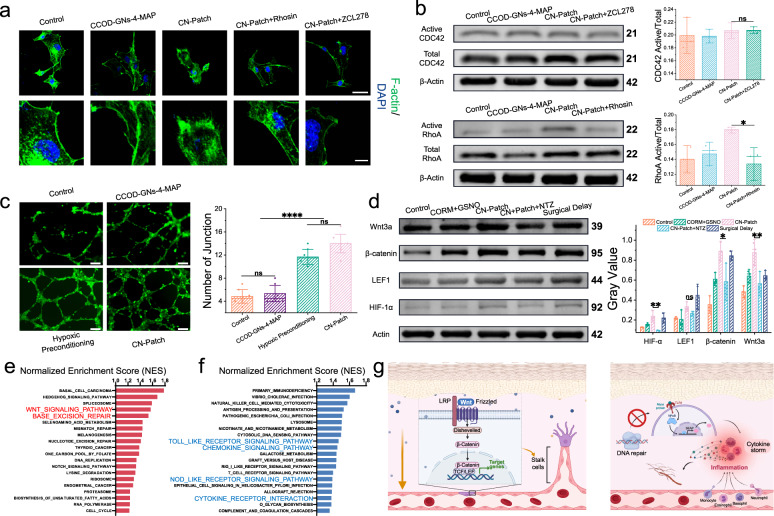
The CN-Patch improves transplant survival by promoting angiogenesis and reducing inflammatory responses. **a** The CN-Patch increased the F-actin level of HUVECs, but this process was weakened by Rhosin and ZCL278. Scale bar: 40 µm, 10 µm from top to bottom (*n* = 6). **b** The Pull-down technology was used to detect the activation of Rho GTPase by CN-Patch. The CN-Patch has a significantly activating effect on RhoA-22kDa, but not on CDC42-21kDa (mean ± SEM, *n* = 3 independent samples per group). **c** The angiogenesis experiment: Compared to the control group, the CN-Patch promoted HUVECs vascular formation in vitro, which was consistent with the hypoxic preconditioning group. Scale bar: 100 µm (mean ± SEM, *n* = 6 independent samples per group). **d** The Western Blot analysis showed that the expression of Wnt/β-catenin pathway-related proteins in the skin flap increased on 7th day after transplantation in rats. After treatment with the CN-Patch and the surgical delayed, the expression of Wnt/β-catenin pathway-related proteins was upregulated, and the effect of CN-patch was better (Wnt3a-39kDa, β-catenin-95kDa, LEF1-44 kDa, HIF1α-92kDa) (mean ± SEM, *n* = 3 independent samples per group). **e** The top 20 most significantly upregulated pathways based on KEGG pathway analysis had a GSEA enrichment score. **f** The top 20 most significantly downregulated pathways based on KEGG pathway analysis had a GSEA enrichment score. **g** The diagram of the possible molecular mechanisms underlying the CN-Patch in reducing inflammation and promoting tissue repair. This picture was designed using BioRender graphic tool (BioRender.com). Error bars indicate Standard Error. The horizontal line indicates the Median Value. Source data and exact *p* values are provided in the Source data file.

Endothelial cell migration is a critical component of angiogenesis. Studies have demonstrated that NO can modulate the function of vascular endothelial cells by regulating the expression of GTPases. Following a 30-min CN-Patch stimulation, HUVECs exhibited a significant increase in stress fiber F-actin cilia, thickening of intracellular stress fiber bundles, and formation of pseudopodia. This effect was attenuated by the Rho GTPases inhibitors Rhosin and ZCL278 (Fig. 6a).

Further, we used the pull-down technology to detect the activation of Rho family proteins RhoA and CDC42 in each group. The CN-Patch had a significant activation effect on RhoA, but was not sensitive to the activation effect of CDC42 (Fig. 6b)^37,38^. We speculated that the CN-Patch may affect the cytoskeleton through GTPases to promote endothelial cell migration. The use of HUVECs in an ischemia-hypoxia-reperfusion injury model in vitro proved that the CN-Patch can effectively promote angiogenesis in vitro, and its effect was not weaker than that of ischemic preconditioning (Fig. 6c). The Western Blot analysis showed that the expression of Wnt/β-catenin pathway-related proteins increased in rat skin flaps on 7th day after transplantation. After the CN-Patch and surgery delay treatment, the expression of Wnt/β-catenin pathway-related proteins was upregulated, and the effect of the CN-Patch treatment was more evident (Fig. 6d).

We next performed high-throughput genome-wide RNA-seq on the compression zone tissue from the CN-Patch and control groups at 7 d after surgery. As shown in Supplementary Fig. 14, gene expression analysis revealed 828 differentially expressed genes (*p* < 0.05) between the CN-Patch and control groups, with 399 upregulated genes(such as CISH, Ptch, RT1BB) and 429 downregulated genes (such as CTSS, CCL20, NR1D1, EGR1). Gene set enrichment analysis (GSEA) of the biological functions of the upregulated genes found that they were enriched in pathways that promoted cell adhesion, proliferation, differentiation, and DNA damage repair. The representative signaling pathways included the WNT signaling pathway and genome sequence repair pathway (Fig. 6e)^39,40^. Further analysis of these representative signaling pathways revealed more related genes, including ERCC1 and WNT3, which were significantly upregulated in the CN-Patch group. ERCC1 is essential for repairing DNA damaged by hypoxia-ischemia, while WNT3 is an important ligand in the WNT signaling pathway. The regulation of WNT signaling in endothelial cells is necessary for neovascularization^41,42^. β-catenin, a molecule downstream of the WNT signaling pathway, exists both intracellularly and extracellularly, and it can help the transmembrane adhesion protein VE-cadherin bind to the cell membrane, thereby increasing the stability of the cytoskeleton and supporting blood vessel generation/maturation processes^43^.

Multiple inflammation-related pathways were downregulated in the CN-Patch group, including the Toll-like receptor signaling pathway, the chemokine signaling pathway, the cytokine-cytokine receptor interaction, and the NOD-like receptor signaling pathway (Fig. 6f). The ischemia-reperfusion injury activates the inflammatory process in the flap tissue, leading to the release of a large number of pro-inflammatory chemokines and cytokines. The transcription factor NF-κB is a key regulator of vascular inflammatory responses, inducing the downstream transcription of inflammatory genes, such as the pro-inflammatory molecules TNF-α, IL-6, and IL-1β^44^. The effective reduction of NF-κB production by the CN-Patch may be the basis of the suppression of the inflammatory storm. Immunohistochemical analysis and real-time fluorescent PCR detection of the choke area tissue verified that the CN-Patch group had a lower inflammatory response compared with the control group and the surgery-delayed group, which may benefit from the CO-released protection of CN-Patch (Supplementary Fig. 15).

Overall, we speculated that the main mechanism of the CN-Patch in improving the survival rate of transplanted tissues included the long-term release of NO in the perioperative period to activate the Wnt pathway, thereby regulating the activation and migration of endothelial cells and promoting angiogenesis after injury (Fig. 6g). Additionally, the CN-Patch helped to maintain early blood supply and reduce ischemia-reperfusion injury, thereby inhibiting inflammatory injury.

## Discussion

Flap transplantation has become a popular method for repairing large soft tissue defects, however, hemodynamic and ischemia-reperfusion injury often leads to unavoidable local necrosis or complete reconstruction failure. These disturbances can occur not only in the vascular pedicle, but also in skin valve tissue microcirculation, making conventional surgery, systemic anticoagulants, and antispasmodics ineffective. To address the inflammatory storm caused by free radical oxygen overload and ischemia-reperfusion, various drug delivery systems have been developed, yet no investigation exists to repurpose an optimal drug delivery system in the field of flap transplantation.

We have proposed a local CO and NO gas molecules release-on-demand system (CN-Patch), which can reduce IRI, promote continuous blood supply, and improve the microcirculation of the transplanted tissue repair. The CN-Patch acts as a direct vasodilator, which is involved in various hypoxia/ischemia-induced biological processes. Specifically, the CN-Patch increased the survival rate and promoted functional recovery of free flaps, mainly by reducing endothelial inflammatory storm and promoting neovascularization. In the early postoperative period, IRI causes increased production of free radicals and inflammatory factors, and induces intracellular calcium overload, DNA fragmentation, thrombosis, inflammatory response, and apoptosis. The timely release of CO and NO activates signaling pathways, such as MAPK, FOXO, and PI3K, strongly inhibiting platelet aggregation and endothelial cells apoptosis, and significantly blocking the positive feedback loops between TNF-α, IL-1β, and NF-κB, thereby efficiently maintaining endothelial cell integrity. The sustained release of NO after transplantation surgery positively regulates endothelial cell proliferation, vessel remodeling, and angiogenesis through various pathways such as the WNT signaling pathway, while consistently improving microcirculation perfusion. Postoperative release cycles for more than 7 days maintained adequate total donor blood flow and helped the graft survive the risky period of postoperative blood supply disruption. Furthermore, the CN-Patch expanded the scope of blood supply for single-vessel transplant donors, providing more options for the reconstruction of large soft tissue defects.

Previous studies have reported that secondary budding of the neural plexus and the WNT/β-catenin signaling pathway both play important roles in neural tube development, which may explain the long-term ameliorative effects of the CN-Patch on flap nerve repair^45,46^. Currently, tissue reconstruction and repair surgery strategies aim for “full-scale reconstruction”; which includes not only short-term transplant survival and wound coverage, but also long-term functional reconstruction^47^. Moreover, the CN-Patch group showed almost complete re-innervation of blood vessels after the operation, which is essential for the full function of flap blood vessels and temperature regulation. Re-innervation is critical for long-term functional and sensory recovery after skin flap transplantation^48–50^. Therefore, the CN-Patch has great potential to improve long-term repair in skin flap tissue transplantation, patient prognosis, and quality of life.

As a dual gas spatiotemporal release system in line with the physiological repair process, the CN-Patch has several key advantages: safe gas dosage, immediate local and precise intraoperative drug delivery, and multi-channel full perioperative intervention. This system provides an innovative strategy for improving the survival rate of skin flaps and other organ transplants.

## Methods

### Materials

Chitosan (degree of deacetylation≈95%, 100–200 mps), dextran (Mw: ~70 kDa), 3, 4-dihydroxy hydrocinnamic acid, 1-ethyl-3-(3-dimethylaminopropyl)-carbodiimide hydrochloride (EDC), lead-oxide (ZnO) were purchased from Aladdin. Sodium periodate (NaIO_4_) was purchased from Acros. 4-(Methylamino) Pyridine was purchased from TCI. Fibrin glue was purchased from Shanghai yuanye Bio-Technology Co., Ltd. Gelatin type A (from porcine skin, 300 Bloom), Live/Dead Cell Double Staining Kit, CORM-3, and GSNO were purchased from Sigma. CCK-8 was purchased from Beyotime. CD31, IL-6, TNF-a, IL-1b, and all other antibodies are purchased from Abcam. IR@dye 680-NHS ester and IR@dye 800-NHS ester were purchased from LI-COR.

### Preparation of GNs and GNs-4-MAP

GNs were prepared using a typical two-step desolvation method^29^. Briefly, the gelatin (1.25 g) was dissolved in deionized water (25 mL) at 50 °C. After complete dissolution, acetone (25 mL, 4 °C) was added to precipitate the high molecular weight gelatin in the solution. The supernatant was discarded followed by adding deionized water (25 mL) to redissolve the precipitated gelatin at 50 °C. The pH of the gelatin solution was then adjusted to 2.5 using a hydrochloric acid solution, and then added dropwise acetone (80 mL) to gelatin solution (≈ 4 mL min^−1^) under vigorous agitation. The glutaraldehyde (740 µL, 25 wt%) was added to the GNs suspension. After cross-linking for 16 h, 100 mL of 0.1 M glycine solution was added to terminate the reaction. After centrifuge thoroughly, GNs were lyophilized for further use.

GNs-4-MAP was synthesized through the classical solution saturation lyophilization method^30^. A 100 mg/mL 4-(Methylamino) pyridine aqueous solution was prepared, and the GNs were dispersed in it under an ultrasonic environment, after 1 h ultrasonic treatment, the solution was shaken overnight on a shaker. The reaction solution was first freeze-dried, then fully washed, and subjected to a second freeze-drying to obtain GNs-4-MAP for use.

### Synthesis of Chi-C and Odex

Firstly, chitosan was dissolved in HCl (pH = 4.5). An amount of hydrocinnamic acid and EDC were added to the above solution, and vigorously stirred at room temperature for 7 h while retaining the pH value of 5. After the reaction was completed, the resulting solution was dialyzed in acidified DDW and DDW to remove excess reagents, and Chi-C was obtained by lyophilization. Odex was synthesized by reacting dextran (DEX) with sodium periodate. In short, DEX (4 g) was dissolved in DDW (320 mL) and sodium periodate (4.2 g in 80 mL DDW) solution was added to the above solution. After stirring for 24 h, the resulting solution was dialyzed in DDW (molecular weight cut-off: 12000) and Odex was obtained by lyophilization.

### Formation of the CCOD-GNs-4-MAP and the CN-Patch

Chi-C and Odex were separately dissolved in PBS, and mixed to form CCOD. A mass percentage of GNs-4-MAP was added to the Odex solution, and this solution was mixed with the Chi-C solution to obtain CCOD-GNs-4-MAP. The preparation method of the CN-Patch was similar to that of CCOD-GNs-4-MAP. To achieve the CO and NO gases release on demand, first, Chi-C and Odex were separately dissolved in PBS. A mass of GNs-4-MAP was dispersed into Odex solution, then different concentrations of GSNO were added to this solution, this was solution A. Different concentrations of CORM-3 were added to Chi-C solution, this was solution B. Solution A and solution B were extruded through a screw needle to obtain CN-Patch (A to B volume ratio is 1:1). The preparation process of the CN-Patch was shown in Fig. 2a.

### FT-IR spectra of the samples

All samples were lyophilized, mixed with KBr, and ground into a fine powder. The powder was tested with a BLUCK spectrophotometer within 500–4000 cm^−1^.

### Injectability, self-healing, morphology, and rheological properties of the hydrogels

The prepolymer CN-Patch was added into a single-channel, then was extruded (Diameter: 29 G). The cylindrical hydrogel which was dyed into different colors was cut into two pieces. Then it was pieced together under certain pressure. After 3 minutes, the self-healing ability of the hydrogel was observed.

The microstructure of GNs, GNs-4-MAP, CCOD, CN-Patch, and bacteria was lyophilized and a layer of 2 nm thick Au was sputtered on the surface of the sample to increase its conductivity. The microstructure was observed by the JEOL FESEM 6700 F electron microscope, and the energy of primary electron was 3 kV.

The cylindrical hydrogel was prepared with a 30 mm diameter and 4 mm height. In all the experiments, the parallel plate geometry diameter (25 mm), temperature (25 °C), frequency (0.1 Hz), and oscillatory stress (1 Pa) were kept constant. The self-healing property of the hydrogel was tested by the rheological test in continuous step strain measurements (a fixed frequency of 10 rad s^−1^. Each strain interval was kept as 150 s.).

### Swelling and degradation of the hydrogels in vitro

A certain quality of hydrogel was placed in a 9 cm petri dish with PBS, and the hydrogel was removed from the petri dish and weighed after the excess surface water was removed. The swelling rate (SR) was determined by the following formula^27^:

1 $${SR}\,\left(\%\right)=\frac{(W_t-W_0)}{W_0}\times 100\%.$$

Degradation rate (DR) was determined by the following formula:

2 $${DR}\,\left(\%\right)=\frac{(W_0-W_t)}{W_0}\times 100\%.$$

W_t_ was the weight of the hydrogel taken out of the petri dish at different time points, and W_0_ was the initial weight of the wet hydrogel. The experiment was repeated three times.

### Adhesive strength, hemostatic and antibacterial ability of the hydrogels

Fresh pig skin was used to test the adhesion between the hydrogel and host tissue. The skin tissue was fully washed and simply cut into a 20 × 40 mm^2^ rectangle. 1 mL hydrogel solution was applied to the surface of the pig skin and the other skin was buttoned up (Fig. S3a). The adhesion area was 20 × 10 mm^2^ and tested with a tensile machine (*n* = 3).

The Sprague-Dawley (SD) rats (male, weight 240–280 g) were used to study the hemostatic properties of the hydrogels. The rats were anesthetized with isoflurane and placed on a tilted board (Fig. S3b). The rat liver was exposed through an abdominal incision, and the tissue fluid near the liver was wiped with gauze. A pre-weighed filter paper was placed under the liver, then a 2 mm wound of the liver was cut and the prepolymer solution was immediately injected. After 10 min, the weight of the blood-sucking filter paper in each group was compared with the control group (no treatment after the liver cut).

### CO and NO release study in vitro

The CN-Patch block (1 mL, CORM 200 μM, GSNO 400 μM) was immersed in 3 mL PBS, then 50 μL aliquots of sample solution were consecutively removed at various time points and the soaking solution was replaced to simulate tissue fluid flow. The retardation curves were calculated based on the corresponding kit standard curve using a UV–vis spectrophotometer. All reagents used and the test environment were pre-saturated with high-purity nitrogen.

### Animals

Male Sprague Dawley rats (weight, 280–300 g, 8 weeks old) were received from Beijing Weitong Lihua Biotechnology Co., Ltd. The animals were hosted in equipped animal facility with ambient temperature of 23 °C and humility at 45–55%, under a dark/light cycle of 12 h. Only Male Sprague Dawley rats were used to establish the animal model in our experiments. All animal experimental operations were in accordance with the specifications of the Guide for the Care and Use of Laboratory Animals, and all experimental procedures and protocols were approved by the Animal Experimentation Ethics Committee of Jilin University. (Approval No. 20200677).

### The dyes release in vivo

To predict drug release, the IR@dye 680-NHS ester and IR@dye 800-NHS ester were used to simulate CORM-3 and GSNO (Fig. S8). The gel preparation process was consistent with the CN-Patch, except that CORM-3 and GSNO were replaced with IR@dye 680-NHS ester and IR@dye 800-NHS ester. Nude Mice were anesthetized with isoflurane. 100 µL dye solution or simulated gel were injected into mice’s backs subcutaneously (The volume ratio of the two dyes was 1:1, and the concentration of each dye was 10 mM), followed by a continuous whole body imaging (LI-COR Odyssey Mouse POD) for 14 d.

### CO and NO release study in vivo

36 adult male SD rats (weight, 280–300 g) were randomly divided into the normal group, CORM + GSNO group, and CN-Patch group. Briefly, Equal volumes of PBS, CORM + GSNO PBS solution, and newly synthesized CN-Patch were respectively injected in the caudal choke area. Blood samples were collected from the femoral artery at the designated time points after injection (1 h, 12 h, 72 h, and 168 h) and carefully stored for the detection of CO and NO. After blood collection, the caudal choke area (1 × 3 cm^2^) was collected. The NO concentration of serum and tissue were analyzed using an NO assay kit. Another part of the collected blood was used for measuring carboxyhemoglobin saturation (HbCO %) according to the corresponding formula.

### Degradation of the CN-Patch in vivo

To better track the degradation of the CN-patch, a 1.5 × 2 cm^2^ rectangular skin flap was prepared on each rat’s dorsum, and 500 μL of CN-Patch was implanted subcutaneously. Flap pockets were opened at different time points (1, 3, 9, 14, 17, 21, 24, and 28 d). The remaining hydrogel under the flaps was photographed at each time point.

The fluorescence degradation tracking of the CN-Patch was similar to the dyes release in vivo, with IR@dye 800-NHS ester grafted onto chitosan and GNs backbones.

### Infrared thermal thermal imaging

A total of 30 adult male SD rats (weight, 280–300 g) were randomly divided into five groups (*n* = 6), including CORM PBS solution (200 µM, 500 µL per choke zone), GSNO PBS solution (400 µM, 500 µL per choke zone), CORM + GSNO PBS solution (CORM 200 µM, GSNO 400 µM, 500 µL per choke zone), CCOD-GNs-4-MAP (500 µL per choke zone), and the CN-Patch (CORM 200 µM, GSNO 400 µM, 500 µL per choke zone) groups. All rats were anesthetized by continuous inhalation of isoflurane and their dorsal hair was carefully removed. Temperatures and thermographic images of rat dorsal areas were acquired using a FLIR infrared camera (T650sc, USA).

### Postmortem arteriography

To further investigate vasodilatation function, 24 rats were randomized into control, CORM + GSNO PBS solution, CCOD-GNs-4-MAP, and CN-Patch groups, which underwent the same injection procedures described above. The rats were euthanized and perfused with lead-oxide gel through the aorta. Then, rat bodies were transferred to a 4 °C refrigerator and stored for 24 h. Flaps were harvested and examined using a Siemens X-ray machine (Siemens, Germany) to reveal their vascular networks.

### Ischemia-reperfusion dorsal skin flap model of rats

A total of 60 adult male SD rats (weight 280–300 g) were randomized into five groups, as follows: control, CORM + GSNO, CCOD-GNs-4-MAP, surgical delay, and CN-Patch. All rats were anaesthetized by isoflurane inhalation, and their backs were shaved and disinfected. A rectangular 11 × 3 cm^2^ single pedicled island flap was then made on one side of the back and flaps were only connected to the body by the deep circumflex iliac artery. CCOD-GNs-4-MAP or CN-Patch was applied onto the inner surfaces of the two choke zones. After curing, the only perforator was occluded using a microvascular clip. Clips were released 5 h later, and flaps were sutured in situ using 4-0 nylon. For the CORM + GSNO group, the solution was subcutaneously injected into each choke zone. For the surgical delay group, 5 d before the flap operation, a 3 cm longitudinal incision was made in the back of the rat, the perforator of the posterior intercostal artery was ligated and the flap was sutured in situ. The surgical delay procedure was followed as Supplementary Fig. 9c.

### NIR-II angiography

The groups were as follows: Control, CORM + GSNO, CCOD-GNs-4-MAP, Surgical delay, and CN-Patch (*n* = 6). Rats were anesthetized by isoflurane inhalation and the hairs on the back areas were shaved. Anesthetized rats were placed in a prone position and injected with IR-780@BSA probe (6 mM, 3 ml/kg) via tail vein^51,52^. All NIR-II images were obtained within 3 min after injection of the probe, using a two-dimensional InGaAs camera (Princeton Instruments, NIRvana-640). Images were collected with an 1100 nm long pass filter and 40 ms exposure time, under an 808 nm laser excitation (65 mW/cm^2^). To maximize image quality, each flap image was pieced together from three segments: anterior, mid, and posterior parts using ImageJ software (ImageJ v.1.52a) and Photoshop (Adobe, CC2018).

### Laser speckle imaging

The flap blood flow was continuously monitored using a laser speckle imaging system (RWD RFLSI III). Rat back flaps were lifted up and only connected to the body by the deep circumflex iliac artery. A laser speckle imaging system was used to assess blood flow and record images.

### Assessment of long-term neurological function

CTMr tests were conducted to assess the nociceptive sensation of flaps three months post-surgery^35^. Briefly, a pair of forceps was used to pinch three parallel spots in the proximal, middle, and distal parts of the skin flap. Pinching normal dorsum skin with forceps elicited distinct wrinkles, and based on this phenomenon, average reflex intensity scores were calculated for each skin flap by taking the average of scores for the three spots.

### mRNA transcriptome sequencing (RNA‐seq) and bioinformatics analysis

RNA integrity was assessed using an RNA Nano 6000 Assay Kit for the Bioanalyzer 2100 system (Agilent Technologies, CA, USA). Total RNA was used as input material for RNA sample preparation. The obtained data underwent quality control, was mapped to the reference genome, and was used to predict novel transcripts. Gene expression levels were quantified and subjected to differential expression analysis. Additionally, GO and KEGG enrichment analysis, protein-protein interaction analysis, and weighted correlation network analysis of differentially expressed genes, were conducted.

### General procedure for western blotting

Inhibitors of phosphatase and protease (P002, NCM, China) were added to RIPA buffer to lyse the cells and tissues, and total proteins from the cells or tissues were extracted in the supernatant. The protein concentrations of cell samples were measured with BCA Protein Assay Reagent (P0010S, Beyotime, China). Post lysis, proteins were separated by SDS-PAGE (150 V, 60 min) before being transferred to a PVDF membrane (1620177, Bio-Rad, USA) with a Trans-Blot wet transfer (Bio-Rad, 400 mA, 25 min). Blots were then incubated with the primary antibody in fresh blocking buffer at 4 °C overnight. The primary antibody concentrations used were as follows: Anti-HIF-1 alpha antibody (dilution of 1:1000, catalog number: ab179483, clone: ERP16897, Abcam), Anti-beta Catenin antibody (dilution of 1:500, catalog number: ab32572, clone: E247, Abcam), Anti-LEF1 antibody (dilution of 1:500, catalog number: ab137872, clone: EPR2029Y, Abcam), Anti-Wnt3a antibody (dilution of 1:500, catalog number: ab219412, clone: EPR21889, Abcam). After overnight incubation, blots were washed with TBST (WB20500, NCM, China) and then incubated with the dye-labeled IRDye® 800CW Goat anti-Rabbit IgG Secondary Antibody (dilution of 1:15000, catalog number: 926-32211, lot: D00825-14, LI-COR) in fresh blocking buffer at room temperature for 1 hour. Once complete, blots were washed again in TBST. The molecular weight (MW) of each protein shown on immunoblots was estimated based on Pre-Stained Standard (10-180KD) (26616, Thermo, USA). The images of immunoblots were quantified with Image J software 1.8.0. The relative levels of target proteins were normalized by β-Actin (dilution of 1:50000, catalog number:AC026, clone:ARC51105-01, ABclonal).

### Gene expression by quantitative PCR

Total RNA was isolated from rat tissue using TRIzol reagent (T9424, Sigma-Aldrich, USA) according to the manufacturer’s instructions. The gene expression levels were analyzed by real-time quantitative PCR (qRT-PCR). First-strand cDNA was synthesized from 2 μg total RNA in 10 μL reaction mixture using a cDNA synthesis kit (RR036A, TAKARA, Japan) according to the manufacturer’s instructions. Prepared cDNA (2 μL) was used as a template for qRT-PCR. The qRT-PCR was performed using 2×Real Star Fast SYBR qPCR Mix (SYBR Green with ROX;Enzynomics, Genster, China) according to the manufacturer’s instructions. Relative gene expression levels were determined using rat β-actin as an internal standard gene. The gene-specific primers used for real-time RT-PCR analysis are listed in Supplement Table 1. The primers were obtained from Shanghai Bioengineering Co., LTD.

### Arteriovenous anastomosis

The anterior neck area of the rats was shaved and sterilized with 75% medical alcohol. A median incision was made in each rat’s neck, and the common carotid artery and external jugular vein were then separated and isolated. The external jugular vein was cut off and the distal end was ligated. The cardiac end of the external jugular vein was anastomosed to the common carotid artery in an end-to-side fashion using 12–0 nylon sutures. The CORM + GSNO or CN-Patch was applied to the anastomoses using a 1 ml syringe. The wound was then closed carefully with 4-0 silk sutures.

### Measurement of endothelial function of jugular vein

The rats external jugular veins were isolated, and dissected free from adherent tissue. Each vein was cut into 3-mm vein rings and subsequently equilibrated in an oxygenated Krebs-buffer under a tension of 0.8 g in organ chambers for 1 h. The endothelial function of carotid vein was assessed using DMT wire myograph systems (DMT 620 M, Denmark). Vein rings were precontracted using U-46619.

### Cell culture

The human umbilical vein endothelial cells (HUVECs) were purchased from Shanghai Fuheng Biotechnology Co., Ltd. (FH1122, Shanghai, China), and cultured in endothelial cell medium (ECM; ScienCell) with 10% fetal bovine serum (FBS, FB25015, Clark, Australia) and endothelial growth medium supplements. The cells were incubated at 37 °C in a humidified atmosphere of 5% CO_2_, and the medium was changed every two to three days. HUVECs were typically passaged using trypsin (C100C1, NCM, China) to detach the cells from the culture vessel for subculturing. Cells were authenticated by the morphology. All cell lines were negative by mycoplasma testing.

### Cytotoxicity test in vitro

The CN-Patch cytotoxicity was evaluated using HUVECs. Briefly, log-phase HUVECs were seeded in 96-well plates (1 × 10^4^ cells per well) and cultured in 100 µL medium at 37 °C for 24 h. The prepared CN-Patch (200 µL) was soaked in PBS and incubated at 37 °C. Leach liquor was collected at 1, 12, 24, 48, 72, and 128 h to incubate the cells, and 100 µL DMEM media containing 10 µL CCK-8 (C6005, NCM, China) reagent was added to each well. HUVECs were then incubated for a further 2 h at 37 °C. Finally, the absorbance of each group was read at 450 nm using a microplate reader, where absorbance values were directly proportional to cell viability.

To exclude the direct cytotoxic effects of the CN-Patch, we further performed the Live/Dead staining on HUVECs with Calcein-AM/PI (C2015M, Beyotime, China) Double Stain Kit. Freshly prepared CN-Patch (200 µL) was soaked in PBS and incubated in an incubator at 37 °C. The leach liquor was collected to incubate the cells. Cells were collected by centrifugation and resuspended in AM/PI-buffer. The reaction solution was mixed by pipetting and incubated at 37 °C for 30 minutes. Then, the cells were visualized using an inverted fluorescence microscope. Viable cells showed uniform green fluorescence, while the dead ones were stained in red.

### Cell migration capacity test

An in vitro scratch-wound healing assay was used to evaluate the effect of treatments on HUVEC migratory ability. Lines were drawn on the back of 6-well plates using a marker pen, and transverse lines were drawn uniformly at distances of 1 cm between the lines. HUVECs were seeded in the 6-well plates (5 × 10^5^ cells per well) and cultured in the growth medium (DMEM containing no FBS) until reaching confluence.

### Pull-down assay

After reaching 80% density of endothelial cells in the umbilical vein, we administered a drug stimulation for 30 min and then detected the CN-Patch activation of Rho GTPase through a pull-down technique. The protein extraction and quantification were the same as mentioned above. Active RhoA and Active CDC42 were measured using a pull-down assay kit from Cytoskeleton (16118, ThermoFisher, USA). The concentration of the primary antibody as RhoA (catalog number: ab187027, clone: EPR18134, Abcam) was 1:1000, and the CDC42 (catalog number: ab187643, clone: EPR15620, Abcam) antibody concentration was 1:10000. Following overnight incubation at 4 °C, the blots were washed in TBST (WB20500, NCM, China) and then incubated with IRDye® 800CW secondary antibodies in fresh blocking buffer for 1 hour at room temperature. Afterward, the blots were washed again in TBST. The images of the immunoblots were quantified using Image J software 1.8.0, and the relative levels of the target proteins were normalized by β-actin (dilution of 1:50000, catalog number: AC026, clone: ARC51105-01, ABclonal).

### Tube formation assay

In order to examine the effect of CN-Patch on angiogenesis, a Matrigel tube formation assay was performed using HUVECs. The assay was performed according to the manufacturer’s protocol (BD, Endothelial Cell Tube Formation Assay). Briefly, HUVECs were serum-starved in EGM-2 media supplemented with 0.2% FBS for 24 h. 96-well culture plates were coated with 50 μL Matrigel, which was allowed to solidify for 1 hour at 37 °C. The pre-treated HUVECs were then seeded in the well. Meanwhile, the conditioned medium was collected and added to each well. The HUVECs were then incubated at 37 °C for 6 h. The assay plate was incubated at 37 °C for 12 h.

### Stress fiber polymerization assay

We treated the primary cultured HUVECs with different groups of medium for 30 min, stained the cytoskeleton protein F-actin with FITC-labeled phalloidin (CA1620, Solarbio, China), and observed it through a laser confocal microscope.

### Statistical analyses

Data were represented as mean values ± SEM. Statistical analysis was performed with the Prism 8.4/8.5 software (GraphPad Software). Normal distribution was tested by Shapiro-Wilk test. If normality criteria were met, one-way ANOVA with posthoc multiple comparison test was run; if normality criteria were not met, the Kruskal–Wallis non-parametric test was run. (*p < 0.05, **p < 0.01, ***p < 0.001, ****p < 0.0001). The graphics in this manuscript were created using BioRender (https://biorender.com/) and Adobe Illustrator (version 2021).

### Reporting summary

Further information on research design is available in the Nature Portfolio Reporting Summary linked to this article.

## Supplementary information

Supplementary Information

Description of Additional Supplementary Files

Supplementary Movie 1

Reporting Summary

## Data Availability

The RNA-seq data generated in this study have been deposited in the NCBI Gene Expression Omnibus database under accession code “GSE205580”. All other data that support the findings of this study are available at "Figshare [10.6084/m9.figshare.22566988]”, and within the article and the Supplementary Information or from the corresponding author upon reasonable request. Source data are provided with this paper.

## Source data

Source data

## References

[1] Lee GK, Sheckter CC (2018). Breast reconstruction following breast cancer treatment-2018. J. Am. Med. Assoc..

[2] Sorg H, Harder Y, Krueger C, Reimers K, Vogt PM (2013). The nonhematopoietic effects of erythropoietin in skin regeneration and repair: from basic research to clinical use. Med. Res. Rev..

[3] Wang WZ, Baynosa RC, Zamboni WA (2011). Update on ischemia-reperfusion injury for the plastic surgeon: 2011. Plast. Reconstr. Surg..

[4] Fosnot J (2011). Closer to an understanding of fate: the role of vascular complications in free flap breast reconstruction. Plast. Reconstr. Surg..

[5] Gurlek A, Celik M, Parlakpinar H, Aydogan H, Bay-Karabulut A (2006). The protective effect of melatonin on ischemia-reperfusion injury in the groin (inferior epigastric) flap model in rats. J. Pineal Res..

[6] Chen L (2017). Pre-vascularization enhances therapeutic effects of human mesenchymal stem cell sheets in full thickness skin wound repair. Theranostics.

[7] Hendrickx B (2010). Integration of blood outgrowth endothelial cells in dermal fibroblast sheets promotes full thickness wound healing. Stem Cells.

[8] Laschke MW, Menger MD (2016). Prevascularization in tissue engineering: current concepts and future directions. Biotechnol. Adv..

[9] Taylor GI, Corlett RJ, Ashton MW (2017). The functional angiosome: clinical implications of the anatomical concept. Plast. Reconstr. Surg..

[10] Taylor GI, Corlett RJ, Dhar SC, Ashton MW (2011). The anatomical (Angiosome) and clinical territories of cutaneous perforating arteries: development of the concept and designing safe flaps. Plast. Reconstr. Surg..

[11] Taylor GI, Palmer JH (1987). The vascular territories (angiosomes) of the body: experimental study and clinical applications. Br. J. Surg..

[12] Meli EZ (2019). Surgical delay may extend the indications for nipple-sparing mastectomy: a multicentric study. Eur. J. Surg. Oncol..

[13] Chen M, Li X, Jiang Z, Gong X (2019). Visualizing the pharmacologic preconditioning effect of botulinum toxin type A by infrared thermography in a rat pedicled perforator island flap model. Plast. Reconstr. Surg..

[14] Lineaweaver WC (2004). Vascular endothelium growth factor, surgical delay, and skin flap survival. Ann. Surg..

[15] Eltzschig HK, Eckle T (2011). Ischemia and reperfusion-from mechanism to translation. Nat. Med..

[16] Chouchani ET (2014). Ischaemic accumulation of succinate controls reperfusion injury through mitochondrial ROS. Nature.

[17] Giustarinia D, Milzanib A, Colombob R, Dalle-Donneb I, Rossia R (2003). Nitric oxide and S-nitrosothiols in human blood. Clin. Chim. Acta.

[18] Zafonte RD, Wang L, Arbelaez CA, Dennison R, Teng YD (2022). Medical gas therapy for tissue, organ, and CNS protection: a systematic review of effects, mechanisms, and challenges. Adv. Sci..

[19] Ignarro LJ, Buga GM, Wood KS, Byrns RE, Chaudhu G (1987). Endothelium-derived relaxing factor produced and released from artery and vein is nitric oxide. Proc. Natl Acad. Sci. USA.

[20] Carlström M (2021). Nitric oxide signalling in kidney regulation and cardiometabolic health. Nat. Rev. Nephrol..

[21] Otterbein LE (2003). Carbon monoxide suppresses arteriosclerotic lesions associated with chronic graft rejection and with balloon injury. Nat. Med..

[22] Otterbein LE (2000). Carbon monoxide has anti-inflammatory effects involving the mitogen-activated protein kinase pathway. Nat. Med..

[23] Polhemus DJ, Lefer DJ (2014). Emergence of hydrogen sulfide as an endogenous gaseous signaling molecule in cardiovascular disease. Circ. Res..

[24] Yang T, Zelikin AN, Chandrawati R (2018). Progress and promise of nitric oxide-releasing platforms. Adv. Sci..

[25] Wegiel B (2010). Nitric oxide–dependent bone marrow progenitor mobilization by carbon monoxide enhances endothelial repair after vascular injury. Circulation.

[26] Li L, Hsu A, Moore PK (2009). Actions and interactions of nitric oxide, carbon monoxide and hydrogen sulphide in the cardiovascular system and in inflammation-a tale of three gases. Pharmacol. Ther..

[27] Tang X (2021). Magnesium oxide‐assisted dual‐cross‐linking bio‐multifunctional hydrogels for wound repair during full‐thickness skin injuries. Adv. Funct. Mater..

[28] Hu H, Xu FJ (2020). Rational design and latest advances of polysaccharide-based hydrogels for wound healing. Biomater. Sci..

[29] Diba M, Wang H, Kodger TE, Parsa S, Leeuwenburgh SC (2017). Highly elastic and self-healing composite colloidal gels. Adv. Mater..

[30] Munoz Taboada G, Dosta P, Edelman ER, Artzi N (2022). Sprayable hydrogel for instant sealing of vascular anastomosis. Adv. Mater..

[31] Fouquey C, Lehn J-M, Levelut A-M (1990). Molecular recognition directed self-assembly of supramolecular liquid crystalline polymers from complementary chiral components. Adv. Mater..

[32] Chen J (2020). MOFs-based nitric oxide therapy for tendon regeneration. Nanomicro Lett..

[33] Simons M, Gordon E, Claesson-Welsh L (2016). Mechanisms and regulation of endothelial VEGF receptor signalling. Nat. Rev. Mol. Cell Biol..

[34] Liu L, Shi G (2012). CD31: beyond a marker for endothelial cells. Cardiovasc. Res..

[35] Xie Y (2020). Quantification of axonal ingrowth and functional recovery in a myocutaneous flap model in rats with strong clinical implications. Wound Rep. Regen..

[36] Dzau VJ, Braun-dullaeus RC, Sedding DG (2002). Vascular proliferation and atherosclerosis: new perspectives and therapeutic strategies. Nat. Med..

[37] Spiering D, Hodgson L (2011). Dynamics of the Rho-family small GTPases in actin regulation and motility. Cell Adh. Migr..

[38] Dubrac A (2016). Targeting NCK-mediated endothelial cell front-rear polarity inhibits neovascularization. Circulation.

[39] Yousefzadeh MJ (2021). An aged immune system drives senescence and ageing of solid organs. Nature.

[40] Chakrabarti R (2018). Notch ligand Dll1 mediates cross-talk between mammary stem cells and the macrophageal niche. Science.

[41] Stefater JA (2011). Regulation of angiogenesis by a non-canonical Wnt-Flt1 pathway in myeloid cells. Nature.

[42] Low JH (2019). Generation of human PSC-derived kidney organoids with patterned nephron segments and a de novo vascular network. Cell Stem Cell.

[43] Jung Y (2018). Deregulation of CRAD-controlled cytoskeleton initiates mucinous colorectal cancer via beta-catenin. Nat. Cell Biol..

[44] Karunakaran D, Nguyen M, Geoffrion M (2020). RIPK1 expression associates with inflammation in early atherosclerosis in humans and can be therapeutically silenced to reduce NF-κB activation and atherogenesis in mice. Circulation.

[45] Chang CY (2020). WNT3A promotes neuronal regeneration upon traumatic brain injury. Int. J. Mol. Sci..

[46] Eze UC, Bhaduri A, Haeussler M, Nowakowski TJ, Kriegstein AR (2021). Single-cell atlas of early human brain development highlights heterogeneity of human neuroepithelial cells and early radial glia. Nat. Neurosci..

[47] Graney PL (2020). Macrophages of diverse phenotypes drive vascularization of engineered tissues. Sci. Adv..

[48] Hattori Y, Chuang DC, Lan C (2000). Sensory restoration of the skin graft on a free muscle flap: experimental rabbit study. Plast. Reconstr. Surg..

[49] Santanelli F, Tenna S, Pace A, Scuderi N (2000). Free flap reconstruction of the sole of the foot with or without sensory nerve coaptation. Plast. Reconstr. Surg..

[50] Yap LH, Whiten SC, Forster A, Stevenson HJ (2005). Sensory recovery in the sensate free transverse rectus abdominis myocutaneous flap. Plast. Reconstr. Surg..

[51] Tian R (2022). A genetic engineering strategy for editing near-infrared-II fluorophores. Nat. Commun..

[52] Bai L (2022). Super-stable cyanine@albumin fluorophore for enhanced NIR-II bioimaging. Theranostics.

