# Supplementary material for: Exploiting synergistic effect of CO/NO gases for soft tissue transplantation using a hydrogel patch
Source: Nat Commun. 2023 Apr 27;14:2417. doi: 10.1038/s41467-023-37959-y (PMC10140290; doi:10.1038/s41467-023-37959-y)
Supplement: Supplementary file 1 — Supplementary Information [file 41467_2023_37959_MOESM1_ESM.pdf]

Supplementary Information

Exploiting Synergistic Effect of CO/NO Gases for Soft Tissue Transplantation using a Hydrogel Patch

Tang et al.

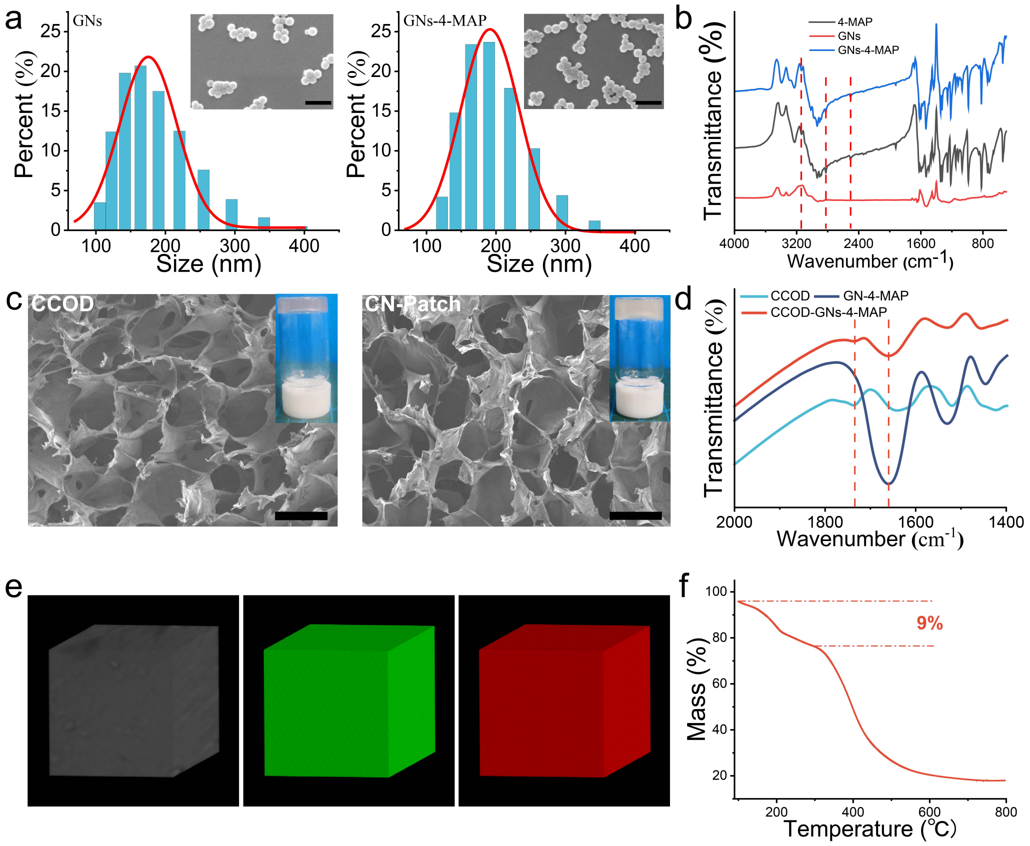

Supplement Figure 1. Characterization of GNs-4-MAP, CCOD-GNs-4-MAP, and CN-Patch.

a, The particle size distributions of GNs, and GNs-4-MAP, the upper inset was the SEM images of the corresponding nanoparticle. The average particle size of the synthesized GNs was about 80 nm. Scale bar: 400 nm.

b, The infrared spectrum of the 4-MAP, GNs and GNs-4-MAP. The infrared spectrum showed that GNs-4-MAP had characteristic peaks of 4-MAP at 3140 cm^{-1}, 2820 cm^{-1}, and 2495 cm^{-1}, these results proved that 4-MAP was successfully loaded onto the GNs.

c, The SEM images of the CCOD and CN-Patch, the inset was the optical images of hydrogels via a tube inverted test. The inverted tube test and SEM images demonstrated that loading with GNs-4-MAP and drug molecule did not affect the gelation rate or the main structure of the gel. Scale bar: 50 μm.

d, The FT-IR spectra of the CCOD, GNs, and CCOD-GNs-4-MAP. The infrared spectrum showed that both CCOD and CCOD-GNs-4-MAP had a characteristic peak of amide bond at 1660 cm^{-1}, which proved successful gelation. CCOD-GNs-4-MAP contained a characteristic peak at 1730 cm^{-1} of GNs-4-MAP, which indicated that GNs-4-MAP was successfully doped into the gel.

e, The 3D fluorescence scanning image. The results showed that GNs could be uniformly dispersed into the gel structure without aggregation. We used the red IR@dye 800-NHS ester to label GNs, and the green IR@dye 680-NHS ester was directly doped into the gel.

f, Thermogravimetric analysis (TGA) thermograms of GNs-4-MAP. The 4-MAP loading rate in GNs-4-MAP was about 9%.

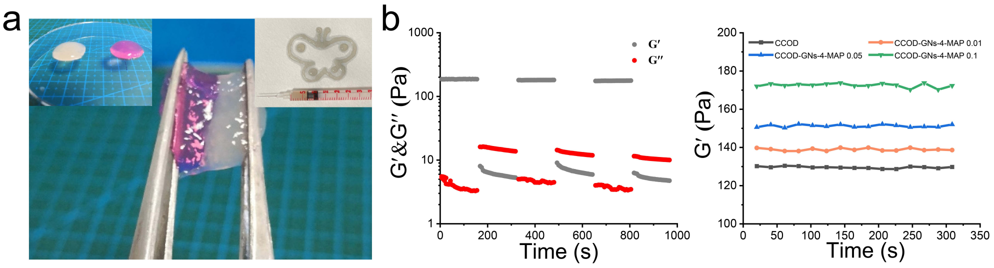

Supplement Figure 2. Characterization of the hydrogel physical properties.

a, The injection and self-healing process of the CN-Patch. The CN-Patch demonstrates successful injection and self-healing. The 29 G needle is able to penetrate the patch, and after incision, the hydrogel fully self-heals without any visible cracks. When hydrogels of different colors are attached together, they are also observed to self-heal completely within 3 minutes without any visible cracks.

b, G′ and G′′ values of the CN-Patch. With the change of shear stress, gel storage modulus, and loss modulus change alternately in continuous step strain measurements (a fixed frequency of 10 rad s^{-1}. Each strain interval was kept as 150 s.), which proved that the doping of GNs-4-MAP did not affect the dynamic self-healing of amide bonds, and was convenient for intraoperative operations. The storage modulus of CCOD-GNs-4-MAP. The rheological results showed that G′ was slightly enhanced with the increase of GNs-4-MAP content, which may be due to the weak interaction between GNs-4-MAP and catechol.

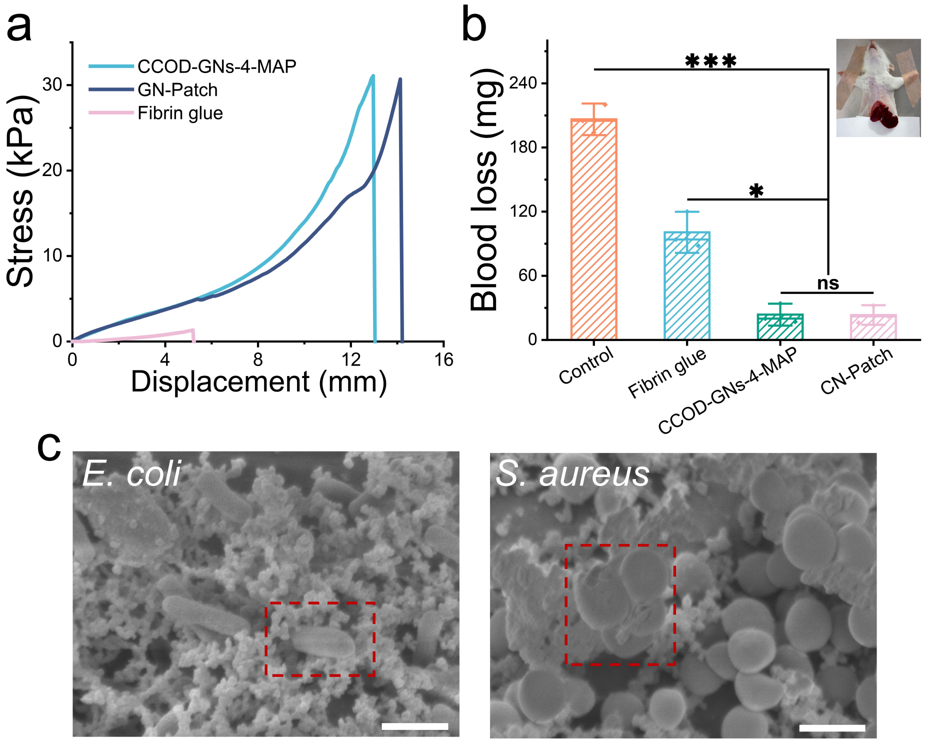

Supplement Figure 3. Characterization of the hydrogel properties.

a. Adhesive strength to porcine skin. The pig skin lap shear test showed excellent real-

time tissue adhesion via dopamine and aldehyde adhesive groups, The adhesion strength of CN-Patch and CCOD-GNs-4-MAP was about 32 kPa, which was about six times greater than commercial fibrin glue.

b. Hemostatic performance of the hydrogels. Assisted by dopamine and aldehyde groups, the blood loss in CCOD-GNs-4-MAP and CN-Patch groups was about 23 mg, while the control group without any intervention was about 204 mg, which demonstrated the gel also exhibited excellent hemostasis. (mean ± SEM, n=3 animals per group). Source data and exact p values are provided in the Source data file.

c. The SEM of *E. coli* and *S. aureus* on the surface of the CN-Patch. The picture in the red frame was the morphology of the dead bacteria. Scale bar: 1 µm. The CN-Patch group could induce apoptosis of *E. coli* and *S. aureus* with the help of Chi-C. (n=3 samples)

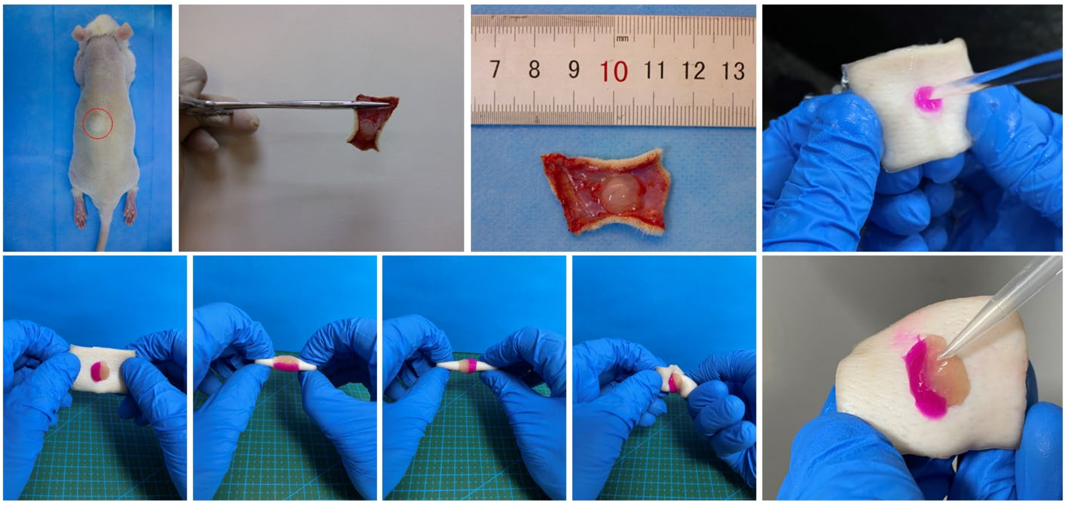

Supplement Figure 4. The CN-Patch adhesion demonstration. The CN-Patch was injectable and could stick to the subcutaneous layer and no detachment after distorting, water-scouring, and blow-drying.

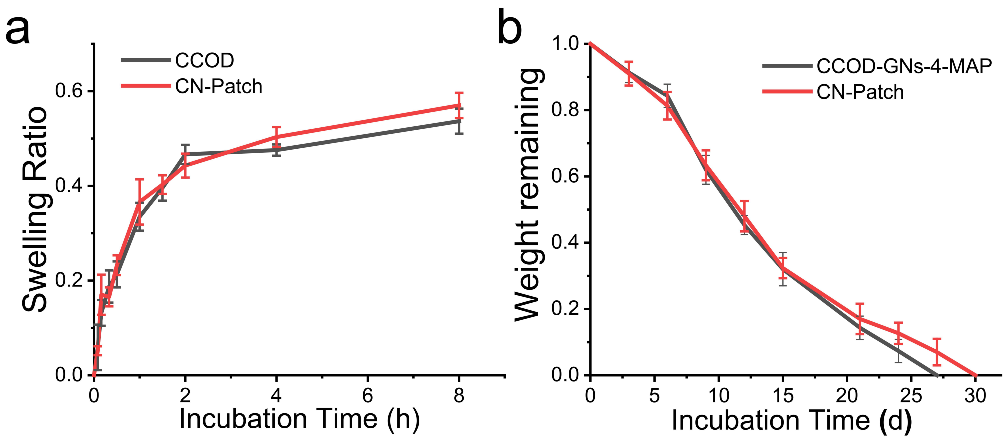

Supplement Figure 5. The hydrogel swelling curve and degradation curve.

a. The CCOD and CN-Patch swelling curve. With the addition of GNs-4-MAP, the gel swelling rate has no obvious change. (mean ± SEM, n=6 samples per group).
b. In vitro and in vivo degradation. The degradation cycle of CN-Patch was about 28 days, which could meet the needs of intraoperative repair (mean ± SEM, n=6 samples per group). Error bars indicate Standard Error.

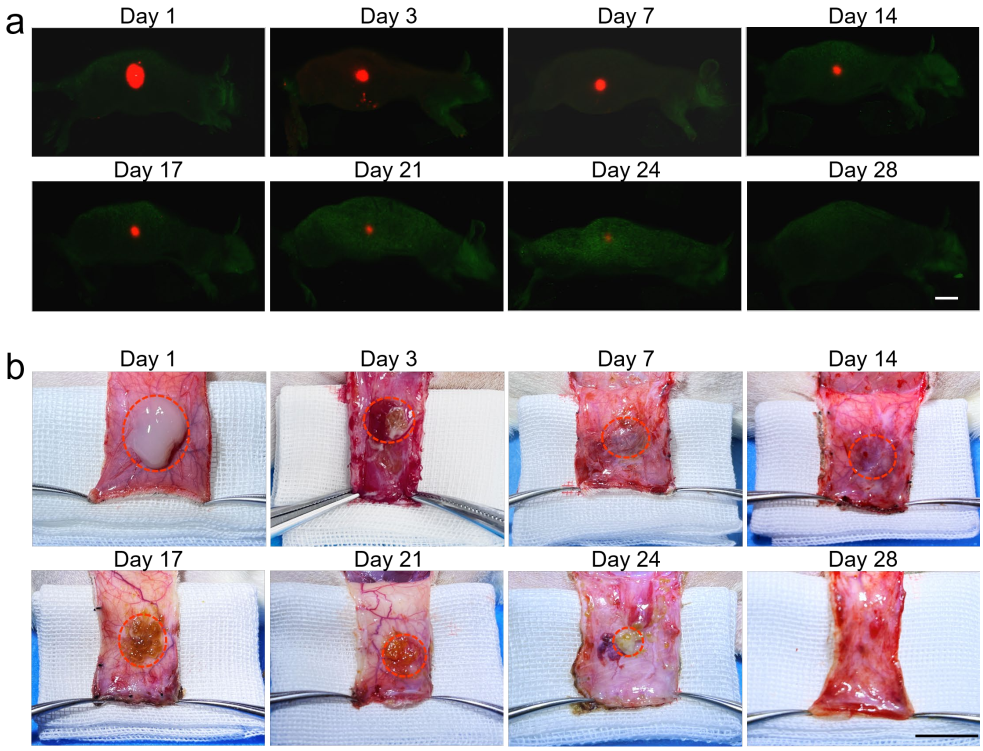

Supplement Figure 6. The image of the CN-Patch hydrogel degradation in vivo.
a, The fluorescence images of the CN-Patch hydrogel degradation. Scale bar: 2 cm. (n=6 animals)
b, The intuitive images of the CN-Patch hydrogel degradation. The degradation period was about 4 weeks (approximately 28 days), which met the requirement of biological function, the red circle represents the outline of residual CN-Patch. Scale bar: 1 cm. (n=6 animals).

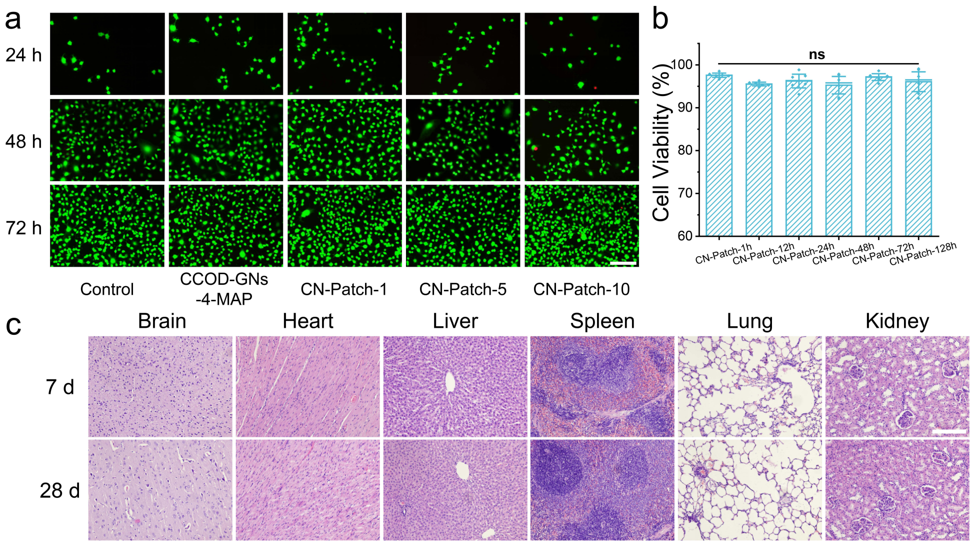

Supplement Figure 7. Bio-compatibility of CCOD-GNs-4-MAP and CN-Patch.

a, The Live/Dead staining of Umbilical vein endothelial cells (HUVECs). Scale bar: 200 µm. (n=3 samples per group)

b, Cytotoxicity analysis of the CN-Patch at different time. (mean ± SEM, n=3 samples per group). Source data and exact p values are provided in the Source data file.

c, The HE staining of the rats' organs. Scale bar:100 µm. The results showed that it had good biocompatibility, and no cell or tissue toxicity. (n=3 animals per group)

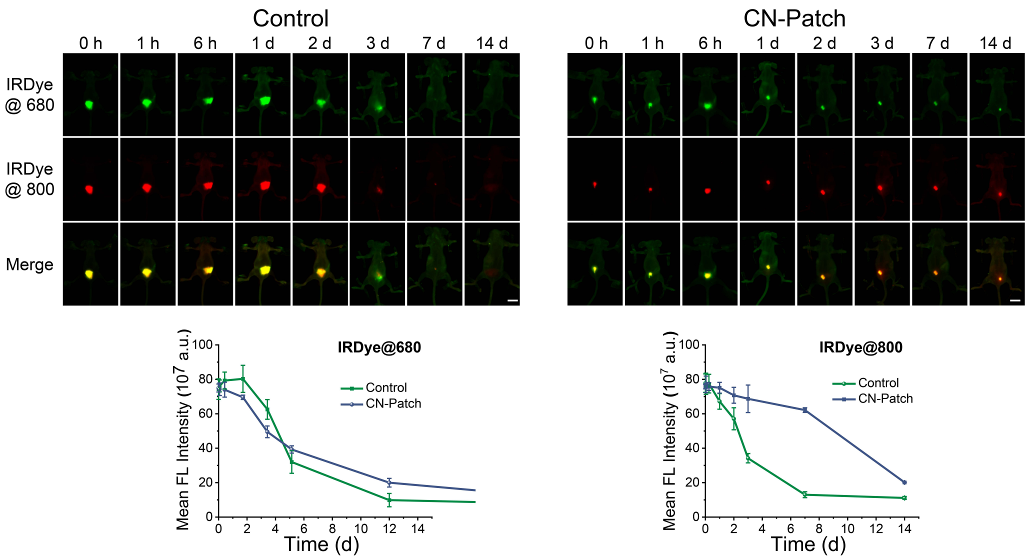

Supplement Figure 8. Fluorescent dyes mimic the release of drug molecules. In order to reduce the dosage and cost of drug molecule preliminary experiments, and to more intuitively determine whether the CN-Patch could facilitate gradient slow-release of CO and NO. We used IR@dye 680-NHS ester and IR@dye 800-NHS ester as prediction model system, and partially grafted IR@dye 800-NHS ester to the surface of GNs through the NHS activation reaction, to simulate whether the capture effect of GNs-4-MAP on NO had the effect of prolonging the sustained release of NO. The sustained-release period of IRDye@680 in the CN-Patch did not significantly change over time, while that of IRDye@800 loaded in the internal GNs was significantly prolonged, these

results are consistent with expectations. Scale bar: 2 cm. (mean ± SEM, n=3 animals per group)

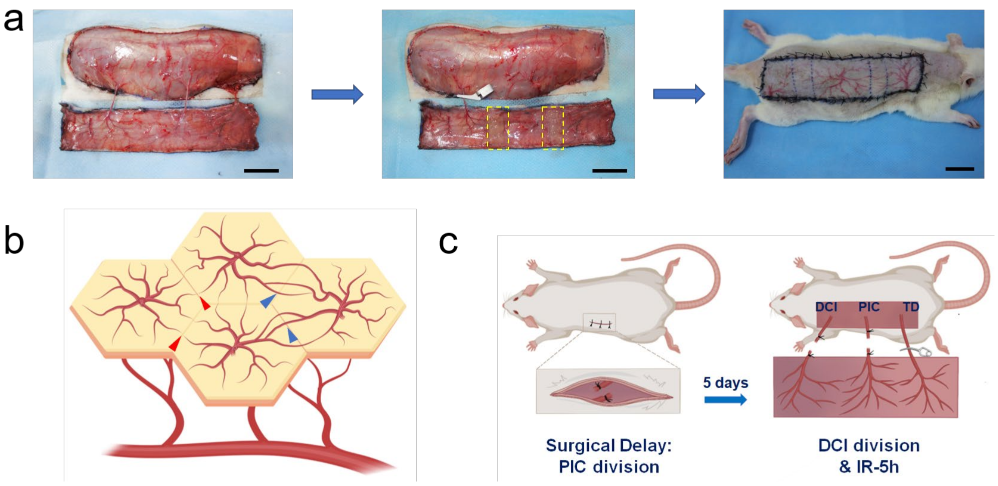

Supplement Figure 9. Surgery flow chart of single pedicle ischemia flap model and the schematic diagram of the angiosome.

a, The surgery flow chart of single pedicle ischemia flap model. Scale bar: 2 cm.

b, Hexagons indicate a single angiosome, a) choke vessels, b) true anastomoses. The angiosome is a three-dimensional block of tissue supplied by a source vessel with its boundary outlined either by an anastomotic perimeter of reduced-caliber choke vessels or by true anastomoses with no reduction of vessel caliber. The illustration was designed using BioRender graphic tool (BioRender.com).

c, The schematic of the surgical delay. The surgical delay was done for five days before the IR operation. A small lateral incision was made in the back. The subcutaneous connective tissue was separated to free the local skin without injuring the vessel. The PIC pedicle was carefully separated, ligated, and cut. The incision was sutured. The flap was then raised according to the method described previously 5 days later. DCI pedicle was ligated and cut. Next, the TD pedicle was isolated by microdissection and clipped using a microscopic vascular clamp, subsequently removed 5 hours later. The illustration was designed using BioRender graphic tool (BioRender.com).

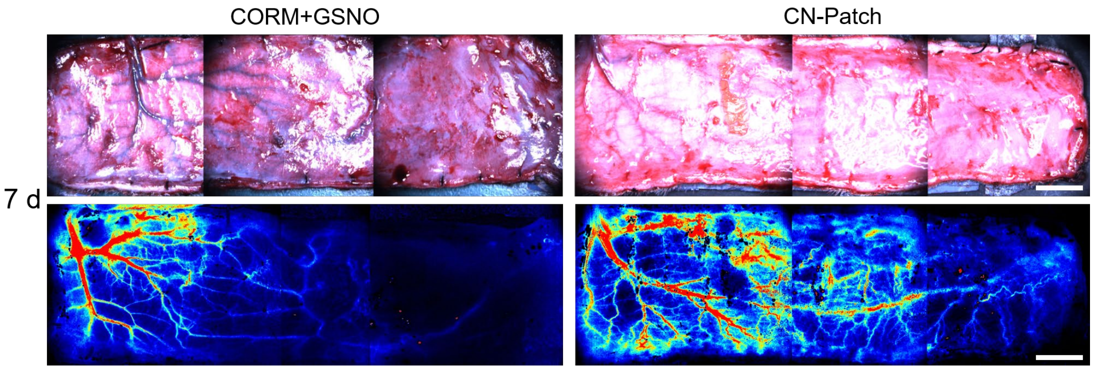

Supplement Figure 10. The macroscopic photographs of inner surfaces of the flaps and laser speckle images of flaps' blood flow 7 days after surgery. Scale bar: 1 cm. (n=3

animals per group)

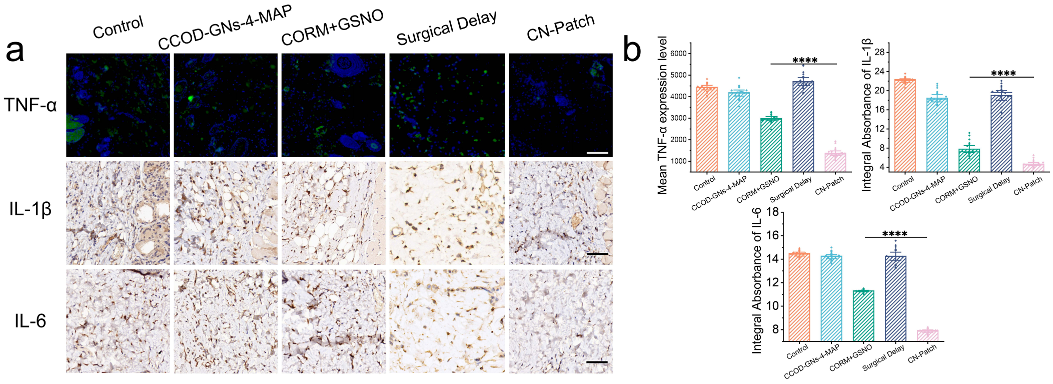

Supplement Figure 11. The inflammation-related immunohistochemical staining of the flaps 7 days after surgery.

a, Distribution of TNF-α (upper row, green), IL-1β (middle row) and IL-6 (lower row) expression of choke zones on day 7 postoperative based on immunofluorescence and immunohistochemistry staining results.

b, Mean TNF-α fluorescence intensity and intergral absorbance of IL-1β, IL-6 of flap choke zones on day 7 postoperative. Scale bar: 50 μm. (mean ± SEM, n=6 animals per group). Error bars indicate Standard Error. The horizontal line indicates the Median Value.

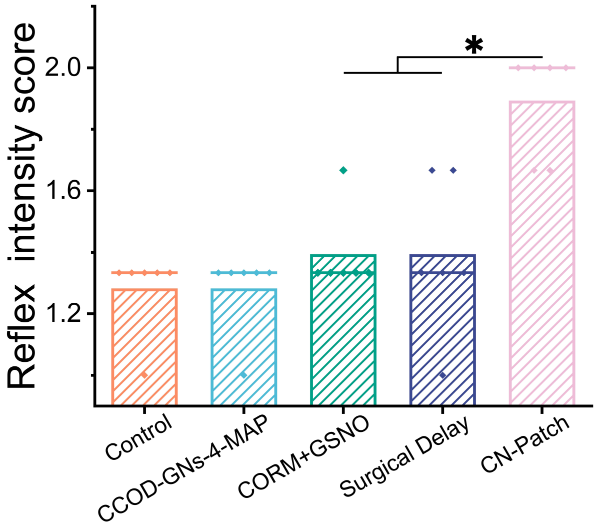

Supplement Figure 12. The cutaneous trunci muscle reflex test. Statistical analysis was performed using a Kruskal–Wallis test followed by a Dunn´s multiple comparisons test (mean ± SEM, n=6 animals per group). Source data and exact p values are provided in the Source data file.

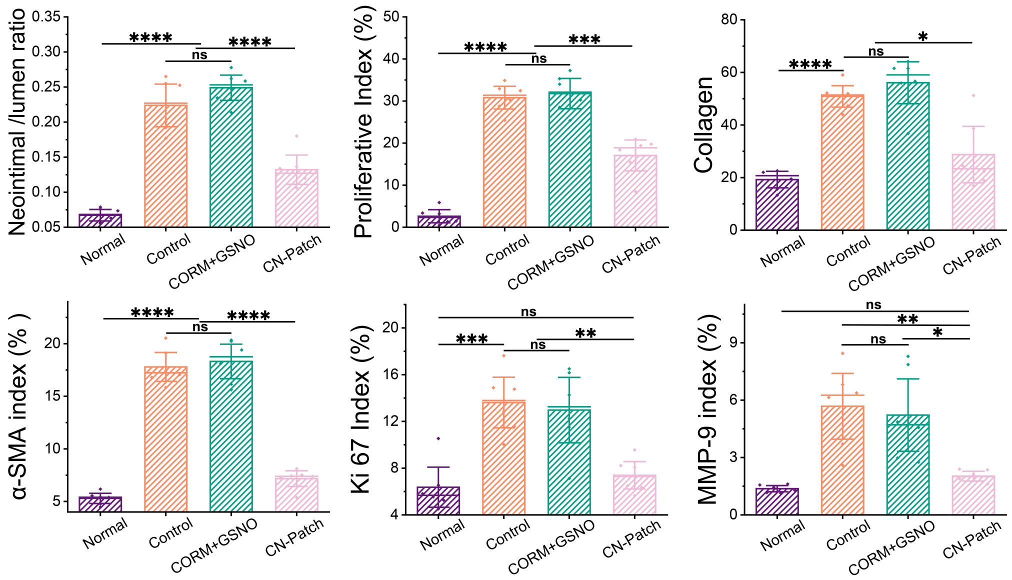

Supplement Figure 13. Histomorphological analysis of jugular vein. (mean ± SEM, n=6 independent samples). Error bars indicate Standard Error. The horizontal line indicates the Median Value. Source data and exact p values are provided in the Source data file.

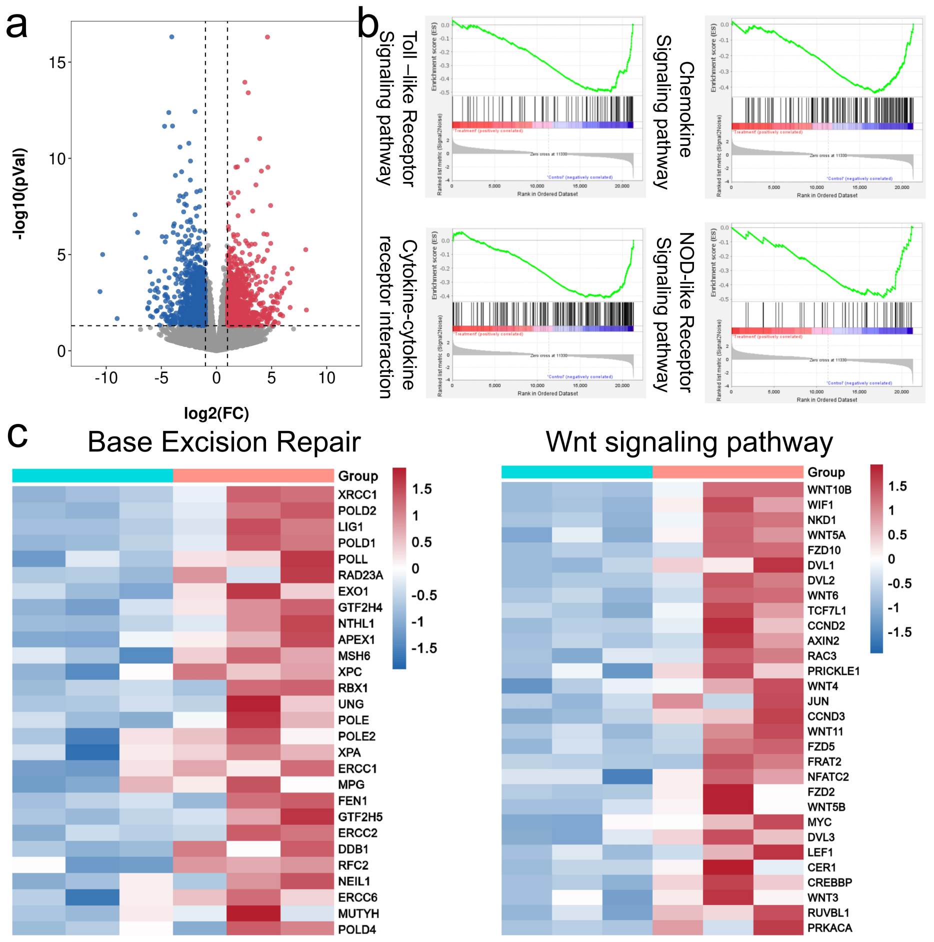

Supplement Figure 14. The mechanism of the CN-Patch based on RNA sequencing. a, The volcano graph showing 828 differential expressed genes between the CN-Patch

group and control group, including 399 upregulated genes and 429 downregulated genes.

b, The 30 most significant down-regulated terms based on GO pathway analysis. The ordinate shows the enriched GO term, and the abscissa stands for the number of genes annotated into GO terms, the color gradient from purple-to-red indicates increasing significance levels.

c, Heatmaps of significantly enriched genes involved in base excision repair (BER) and WNT signaling pathway. The Abscissa is the group name, and the ordinate is the normalized value of the differential gene FPKM. The higher intensities of red mean a higher expression level, while the higher intensities of blue mean a lower expression level.

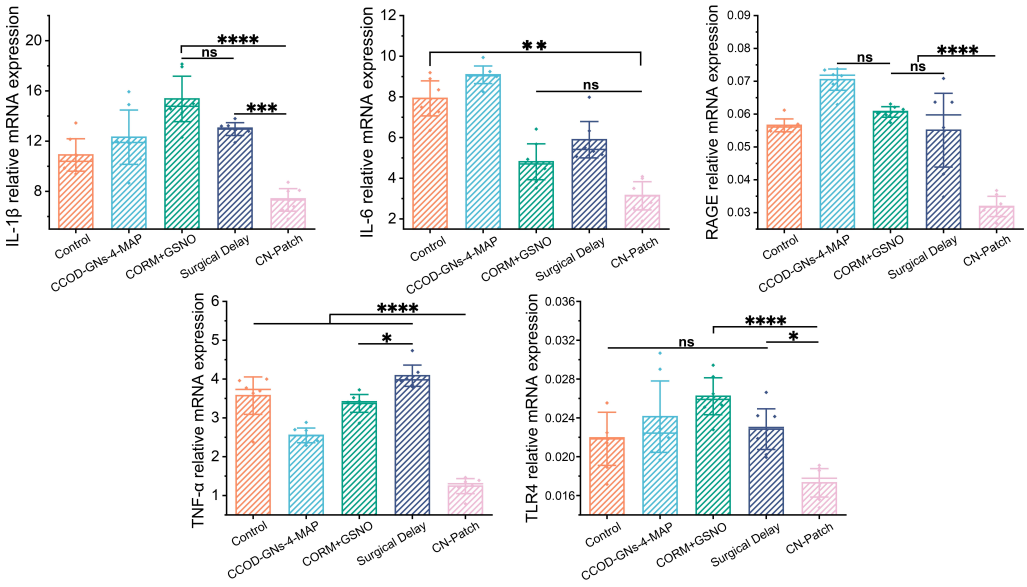

Supplement Figure 15. The RT-PCR testing seven days after a rat transplant revealed expression of inflammatory factors in the skin flap (including IL-6, IL-1β, RAGE, TNF-α, and TLR4). The application of the CN-Patch resulted in a decrease in the expression of inflammatory factors in the skin flap, while the surgical delayed group did not have this effect. (mean ± SEM, n = 6 independent samples). Error bars indicate Standard Error. The horizontal line indicates the Median Value. Source data and exact p values are provided in the Source data file.

TARGET GENE	FORWARD	REVERSE
ACTIN	5'-GGTCAGGTCATCACTATCGGCAATG-3'	5'-CAGCACTGTGTTGGCATAGAGGTC-3'
IL-1B	5'-CTCTTCTCTCCCTCTCTCTTCT-3'	5'-GCCTTGGACTTACAGCTACTT-3'
IL-6	5'-GCCTGCATTAGGAGGTCTTT-3'	5'-CCTGACACCAGCAAAGGATAA-3'
TNF-A	5'-ATGGGAGACGAGGGAGATAAG-3'	5'-CGTCCAACCTCAGCATCTTT-3'
RAGE	5'-CTCACAGCCAATGTCCCTAAT-3'	5'-CTCTCTGTGGAAGGCAACAT-3'
TLR4	5'-CCCTTAGCTGGCAGGATATTT-3'	5'-TCTTAGGAGCCCTGTCTAGTT-3'

Supplement Table 1. Primers used for real-time RT-PCR.
