# Supplementary material for: Exploiting synergistic effect of CO/NO gases for soft tissue transplantation using a hydrogel patch
Source: Nat Commun. 2023 Apr 27;14:2417. doi: 10.1038/s41467-023-37959-y (PMC10140290; doi:10.1038/s41467-023-37959-y)
Supplement: Supplementary file 2 — Description of Additional Supplementary Files [file 41467_2023_37959_MOESM2_ESM.pdf]

<u>Description of Additional Supplementary Files</u>

File name: Supplementary Movie 1

Description: The laser speckle Video of flap choke zones after administration
