# Supplementary material for: Exploiting synergistic effect of CO/NO gases for soft tissue transplantation using a hydrogel patch
Source: Nat Commun. 2023 Apr 27;14:2417. doi: 10.1038/s41467-023-37959-y (PMC10140290; doi:10.1038/s41467-023-37959-y)
Supplement: Supplementary file 4 — Reporting Summary [file 41467_2023_37959_MOESM4_ESM.pdf]

nature portfolio

Corresponding author(s): Junhu Zhang

Last updated by author(s): Mar 9, 2023

Reporting Summary

Nature Portfolio wishes to improve the reproducibility of the work that we publish. This form provides structure for consistency and transparency in reporting. For further information on Nature Portfolio policies, see our Editorial Policies and the Editorial Policy Checklist.

Statistics

For all statistical analyses, confirm that the following items are present in the figure legend, table legend, main text, or Methods section.

n/a	Confirmed	
☐	☒	The exact sample size (*n*) for each experimental group/condition, given as a discrete number and unit of measurement
☐	☒	A statement on whether measurements were taken from distinct samples or whether the same sample was measured repeatedly
☐	☒	The statistical test(s) used AND whether they are one- or two-sided<br>*Only common tests should be described solely by name; describe more complex techniques in the Methods section.*
☒	☐	A description of all covariates tested
☒	☐	A description of any assumptions or corrections, such as tests of normality and adjustment for multiple comparisons
☐	☒	A full description of the statistical parameters including central tendency (e.g. means) or other basic estimates (e.g. regression coefficient) AND variation (e.g. standard deviation) or associated estimates of uncertainty (e.g. confidence intervals)
☐	☒	For null hypothesis testing, the test statistic (e.g. *F*, *t*, *r*) with confidence intervals, effect sizes, degrees of freedom and *P* value noted<br>*Give P values as exact values whenever suitable.*
☒	☐	For Bayesian analysis, information on the choice of priors and Markov chain Monte Carlo settings
☒	☐	For hierarchical and complex designs, identification of the appropriate level for tests and full reporting of outcomes
☒	☐	Estimates of effect sizes (e.g. Cohen's *d*, Pearson's *r*), indicating how they were calculated

Our web collection on statistics for biologists contains articles on many of the points above.

Software and code

Policy information about availability of computer code

Data collection	FT-IR spectra were acquired on a BLUCK spectrophotometer equipment within 500~4000 cm-1.<br>The microstructure of GNs, GNs-4-MAP, CCOD, CN-Patch and bacteria was observed by JEOL FESEM 6700F electron microscope, and the energy of primary electron was 3 kV.<br>Dynamic light scattering measurements were performed at Zetasizer Nano-ZS equipment.<br>The whole mouse body fluorescence intensity was analyzed in vivo imaging device (LI-COR Odyssey Mouse POD).<br>UV-vis spectrum was recorded by a Shimadzu 3100 equipment using quartz cuvettes with an optical path of 1 cm.<br>The storage modulus and loss modulus of the hydrogels were obtained using parallel 25 mm diameter plate geometry by TA Instruments waters LLC equipment.<br>Temperatures and thermographic images of rat dorsal areas were acquired using FLIR infrared camera.<br>Flaps were harvested the following day and examined using a Siemens X-ray machine (Siemens, Erlangen, Germany).<br>Flap blood flow was continuously monitored using a laser speckle imaging system (RWD RFLSI ?).
Data analysis	Origin 8.0, GraphPad Prism 8.4/8.5, King Draw3.0, Excel 2021,Image J 1.52a/1.8.0, Photoshop (Adobe, CC2018), BioRender (https://biorender.com/), Adobe Illustrator (version 2021)

For manuscripts utilizing custom algorithms or software that are central to the research but not yet described in published literature, software must be made available to editors and reviewers. We strongly encourage code deposition in a community repository (e.g. GitHub). See the Nature Portfolio guidelines for submitting code & software for further information.

Data

Policy information about availability of data

All manuscripts must include a data availability statement. This statement should provide the following information, where applicable:

- Accession codes, unique identifiers, or web links for publicly available datasets
- A description of any restrictions on data availability
- For clinical datasets or third party data, please ensure that the statement adheres to our policy

The author declare that the experimental data supporting the findings of this study are available within the article and the Supplementary information Files. Extra data are available corresponding author upon reasonable request. Source data are provided with this paper.

Human research participants

Policy information about studies involving human research participants and Sex and Gender in Research.

Reporting on sex and gender	*Use the terms sex (biological attribute) and gender (shaped by social and cultural circumstances) carefully in order to avoid confusing both terms. Indicate if findings apply to only one sex or gender; describe whether sex and gender were considered in study design whether sex and/or gender was determined based on self-reporting or assigned and methods used. Provide in the source data disaggregated sex and gender data where this information has been collected, and consent has been obtained for sharing of individual-level data; provide overall numbers in this Reporting Summary. Please state if this information has not been collected. Report sex- and gender-based analyses where performed, justify reasons for lack of sex- and gender-based analysis.*
Population characteristics	*Describe the covariate-relevant population characteristics of the human research participants (e.g. age, genotypic information, past and current diagnosis and treatment categories). If you filled out the behavioural & social sciences study design questions and have nothing to add here, write "See above."*
Recruitment	*Describe how participants were recruited. Outline any potential self-selection bias or other biases that may be present and how these are likely to impact results.*
Ethics oversight	*Identify the organization(s) that approved the study protocol.*

Note that full information on the approval of the study protocol must also be provided in the manuscript.

Field-specific reporting

Please select the one below that is the best fit for your research. If you are not sure, read the appropriate sections before making your selection.

☒ Life sciences ☐ Behavioural & social sciences ☐ Ecological, evolutionary & environmental sciences

For a reference copy of the document with all sections, see nature.com/documents/nr-reporting-summary-flat.pdf

Life sciences study design

All studies must disclose on these points even when the disclosure is negative.

Sample size	No statistical method was used to predetermine the sample size. The sample sizes were determined as minimal to lower the cost and be sufficient to obtain statistically significant difference between experimental groups (n=3-6). For property measurement experiments, samples were prepared and tested at least twice. For in vivo studies, each group contains at 3 for evaluating the statistical significance. Fig. 2e, n=6. Fig. 3a, b n=3. Fig. 3d, n=6.Fig. 3f, n=3. Fig. 4b, n=6. Fig. 4d, n=6. Fig. 4f, n=6. Fig. 4g, n=6. Fig. 4h, i n=6. Fig. 5c, n=3. Fig. 5d, e, n=6. Fig. 5f, n=6. Fig. 5g, n=6. Fig. 5h, n=6. Fig. 6a, n=6. Fig. 6b, n=3. Fig. 6c, n=6. Fig. 6d, n=3. Fig. 6a, n=6. Fig. S6a, b, n=6. Fig. S10, n=3. Fig. S8, n=3. Fig. S11, n=6. Fig. S12, n=6. Fig. S13, n=6. Fig. S15, n=6.
Data exclusions	No data was excluder from the analysis.
Replication	All samples were replicated independently for 2-3 times with similar results. For each experiment the statistical analysis is indicated in the figure legends. All attempts at replication were successful.
Randomization	The sample were randomly grouped.
Blinding	The investigators were not blinded to group allocation during data collection and analysis. Analyses in the animal experiments were based on measurements acquired and mostly performed by the same investigator.

Reporting for specific materials, systems and methods

We require information from authors about some types of materials, experimental systems and methods used in many studies. Here, indicate whether each material, system or method listed is relevant to your study. If you are not sure if a list item applies to your research, read the appropriate section before selecting a response.

Materials & experimental systems

n/a	Involved in the study
☐	☒ Antibodies
☐	☒ Eukaryotic cell lines
☒	☐ Palaeontology and archaeology
☐	☒ Animals and other organisms
☒	☐ Clinical data
☒	☐ Dual use research of concern

Methods

n/a	Involved in the study
☒	☐ ChIP-seq
☒	☐ Flow cytometry
☒	☐ MRI-based neuroimaging

Antibodies

Antibodies used

Anti-HIF-1 alpha antibody (dilution 1:1000,catalog number:ab179483,clone:ERP16897,Abcam),
Anti-beta Catenin antibody(dilution 1:500,catalog number:ab32572,clone:E247,Abcam),
Anti-LEF1 antibody(dilution 1:500,catalog number:ab137872,clone:EPR2029Y,Abcam),
Anti-Wnt3a antibody(dilution 1:500,catalog number:ab219412,clone:EPR21889,Abcam),
Anti-RhoA antibody(dilution 1:1000,catalog number:ab187027,clone:EPR18134,Abcam),
Anti-CDC42 antibody(dilution 1:10000,catalog number:ab187643,clone:EPR15620,Abcam),
Anti-β-actin antibody(dilution 1:50000,catalog number:AC026,clone:ARC51105-01,ABclonal),
IRDye® 800CW Goat anti-Rabbit IgG Secondary Antibody(dilution 1:15000,catalog number:926-32211,lot: D00825-14, LI-COR).

Validation

All antibodies were verified by the supplier. The equality data was showed on the manufactures' websites as following
Anti-CD31 antibody
https://www.abcam.cn/cd31-antibody-epr17259-ab182981.html
Anti-VEGF Receptor 2 antibody
https://www.abcam.cn/vegf-receptor-2-antibody-ab2349.html
Anti-alpha smooth muscle Actin antibody
https://www.abcam.cn/alpha-smooth-muscle-actin-antibody-epr5368-ab124964.html
Anti-160 kD Neurofilament Medium antibody [NF-09] - Neuronal Marker
https://www.abcam.cn/160-kd-neurofilament-medium-antibody-nf-09-neuronal-marker-ab7794.html
Anti-IL-6 antibody
https://www.abcam.cn/il-6-antibody-ab208113.html
Anti-IL-1 beta antibody
https://www.abcam.cn/il-1-beta-antibody-rm1009-ab283818.html
Goat Anti-Rabbit IgG H&L (Alexa Fluor® 488)
https://www.abcam.cn/goat-rabbit-igg-hl-alexa-fluor-488-ab150077.html
Goat Anti-Mouse IgG H&L (Alexa Fluor® 488)
https://www.abcam.cn/goat-mouse-igg-hl-alexa-fluor-488-ab150113.html
Anti-TNF alpha antibody
https://www.abcam.cn/tnf-alpha-antibody-epr21753-109-ab205587.html
Anti-Ki67 antibody
https://www.abcam.cn/ki67-antibody-blr021e-ab243878.html
Anti-MMP9 antibody
https://www.abcam.cn/mmp9-antibody-ep1254-ab76003.html

Eukaryotic cell lines

Policy information about cell lines and Sex and Gender in Research

Cell line source(s)	Human umbilical vein endothelial cells (HUVECs) were purchased from Shanghai Fuheng Biotechnology Co., Ltd. (FH1122, Shanghai, China).
Authentication	Cells were only authenticated by the morphology.
Mycoplasma contamination	All cells were tested to be free of mycoplasma contamination.
Commonly misidentified lines (See ICLAC register)	None.

Animals and other research organisms

Policy information about studies involving animals; ARRIVE guidelines recommended for reporting animal research, and Sex and Gender in Research

Laboratory animals	Male Sprague Dawley rats (weight, 280–300 g, 8 weeks old) were received from Beijing Weitong Lihua Biotechnology Co., Ltd. The animals were hosted in equipped animal facility with ambient temperature of 23°C and humility at 45%-55%, under a dark/light cycle of 12 h.
Wild animals	This study did not involve wild animals.
Reporting on sex	Only Male Sprague Dawley rats were used to establish the animal model in our experiments. The sex of animals are generally not considered as a variable, without any significant main effect or interaction involving this factor. Compared to the females SD rats, male rats not affected by periodically changing hormone level, tolerate the surgical procedure better and the male rats models are more stable and reliable.
Field-collected samples	This study did not involve sample collected from the field.
Ethics oversight	All animal experiments were conducted according to the Guide of by Jilin University Animal Care and Use Committee. (Approval No. 20200677).

Note that full information on the approval of the study protocol must also be provided in the manuscript.
